# Light-induced structural changes in a short light, oxygen, voltage (LOV) protein revealed by molecular dynamics simulations—implications for the understanding of LOV photoactivation

**DOI:** 10.3389/fmolb.2015.00055

**Published:** 2015-10-01

**Authors:** Marco Bocola, Ulrich Schwaneberg, Karl-Erich Jaeger, Ulrich Krauss

**Affiliations:** ^1^Lehrstuhl für Biotechnologie, RWTH Aachen UniversityAachen, Germany; ^2^Forschungszentrum Jülich, Institut für Molekulare Enzymtechnologie, Heinrich Heine University DüsseldorfJülich, Germany; ^3^Forschungszentrum Jülich, Institut für Bio- und Geowissenschaften, IBG-1: BiotechnologieJülich, Germany

**Keywords:** LOV domain, photoreceptor, molecular dynamics, signaling, optogenetics

## Abstract

The modularity of light, oxygen, voltage (LOV) blue-light photoreceptors has recently been exploited for the design of LOV-based optogenetic tools, which allow the light-dependent control of biological functions. For the understanding of LOV sensory function and hence the optimal design of LOV-based optogentic tools it is essential to gain an in depth atomic-level understanding of the underlying photoactivation and intramolecular signal-relay mechanisms. To address this question we performed molecular dynamics simulations on both the dark- and light-adapted state of PpSB1-LOV, a short dimeric bacterial LOV-photoreceptor protein, recently crystallized under constant illumination. While LOV dimers remained globally stable during the light-state simulation with regard to the Jα coiled-coil, distinct conformational changes for a glutamine in the vicinity of the FMN chromophore are observed. In contrast, multiple Jα-helix conformations are sampled in the dark-state. These changes coincide with a displacement of the Iβ and Hβ strands relative to the light-state structure and result in a correlated rotation of both LOV core domains in the dimer. These global changes are most likely initiated by the reorientation of the conserved glutamine Q116, whose side chain flips between the Aβ (dark state) and Hβ strand (light state), while maintaining two potential hydrogen bonds to FMN-N5 and FMN-O4, respectively. This local Q116-FMN reorientation impacts on an inter-subunit salt-bridge (K117-E96), which is stabilized in the light state, hence accounting for the observed decreased mobility. Based on these findings we propose an alternative mechanism for dimeric LOV photoactivation and intramolecular signal-relay, assigning a distinct structural role for the conserved “flipping” glutamine. The proposed mechanism is discussed in light of universal applicability and its implications for the understanding of LOV-based optogenetic tools.

## Introduction

Blue-light photoreceptors containing light-oxygen-voltage (LOV) domains regulate a variety of different physiological responses in both eukaryotes and prokaryotes (Demarsy and Fankhauser, 2009; Krauss et al., 2009; Herrou and Crosson, 2011). Their light sensitivity is directly dependent on the photochemistry of a non-covalently bound flavin mononucleotide (FMN) chromophore. Upon illumination with blue light a covalent adduct is formed between the FMN-C4a atom and a strictly conserved cysteine residue in the LOV domain (Swartz et al., 2001). Concomitantly, the FMN-N5 atom becomes protonated with the conserved cysteine likely representing the proton donor (Kennis et al., 2003). In the dark, the FMN-cysteinyl thiol adduct is broken and the FMN-N5 atom deprotonated thus concluding the photocycle. Whereas, adduct formation occurs on a faster (microseconds) timescale (Swartz et al., 2001), adduct decay can take seconds to hours depending on the LOV protein (Zikihara et al., 2006; Jentzsch et al., 2009; Rani et al., 2013; Endres et al., 2015). Most LOV photoreceptors are oligomeric multi-domain sensory systems, consisting of a light-perceiving LOV domain and fused effector domains such as kinases, anti-sigma factors, helix-turn-helix DNA binding domains, phosphodiesterases and cyclases (Möglich et al., 2010; Herrou and Crosson, 2011). Those in turn influence a multitude of different cellular light responses in plants (Möglich et al., 2010), bacteria (Herrou and Crosson, 2011), and fungi (Idnurm et al., 2010). In recent years, it became apparent, that adduct formation leads to small-scale structural changes in the vicinity of the FMN chromophore, which are in many cases relayed to the fused effector domains via helical interdomain linkers (termed N-terminal cap or A'α-helix and C-terminal Jα-helix) (Harper et al., 2003, 2004; Halavaty and Moffat, 2007, 2013; Nash et al., 2011; Diensthuber et al., 2013; Herman et al., 2013; Endres et al., 2015; Herman and Kottke, 2015). There is growing experimental evidence that those structural changes in turn result in altered LOV photoreceptor biological activities (Harper et al., 2004; Vaidya et al., 2011; Aihara et al., 2012; Okajima et al., 2014; Kashojiya et al., 2015).

Based on the modularity of LOV photoreceptors various artificial LOV “photoreceptor” proteins have been constructed in recent years, where light-induced structural changes in the LOV domain have been exploited to allow the control of the biological activity of fused protein domains (for an extensive recent review see Shcherbakova et al., 2015). In most cases, the LOV2 domain of *Avena sativa* phototropin 1 (AsLOV2) was utilized as sensory module in those so-called LOV-based optogenetic tools (Shcherbakova et al., 2015). AsLOV2 represents the best studied and understood LOV domain system. Various complementary biophysical (nuclear magnetic resonance (NMR) and X-ray crystallographic) (Harper et al., 2003, 2004; Halavaty and Moffat, 2007; Yao et al., 2008), mutational (Nash et al., 2008; Zoltowski et al., 2009; Zayner et al., 2012; Zayner and Sosnick, 2014) and functional studies (Harper et al., 2004; Jones et al., 2007; Aihara et al., 2012; Zayner et al., 2012) hint toward A'α and Jα-helix dissociation and/or unfolding as the consequence of light-dependent adduct formation in the protein. In contrast, for the dimeric LOV photoreceptor YtvA of *Bacillus subtilis* and the artificial LOV histidine kinase YF1, constructed using the YtvA LOV domain as sensory domain, a rotational movement of the protruding Jα-helix constituting a coiled-coil like interaction in the dimeric protein, was suggested to cause the observed alteration of effector domain “activity” (Möglich and Moffat, 2007; Möglich et al., 2009; Diensthuber et al., 2013; Engelhard et al., 2013). While the structural consequences of adduct formation are rather well-understood for monomeric AsLOV2, the atomic-level structural rearrangements in the LOV core domain that cause Jα dissociation and unfolding and thus the mode of information flow throughout the protein after photon capture, are not. For dimeric natural and artificial LOV photoreceptors such as YtvA and YF1, the situation is even worse. While several mutational and functional studies support the Jα-helix rotation model (Möglich et al., 2009; Diensthuber et al., 2013; Gleichmann et al., 2013), the associated structural changes have so far not been resolved at the atomic level, due to the lack of X-ray crystallographic data for the full-length proteins in both the light- and dark state. Like for AsLOV2, the mode of information flow between the site of photon capture and the A'α/Jα-helix or even more importantly, to the fused effector domains, remains largely elusive. For the understanding of LOV photoactivation and signaling as well as for the rational design and mutational optimization of recently constructed LOV-based optogentic tools such an atomic level understanding is essential.

This required atomic-resolution information could experimentally be provided by either high-resolution NMR structures of both the dark and light states which are inherently difficult to obtain, or by crystallization of the respective protein under constant illumination, which so far has only been successful in few cases (Vaidya et al., 2011; Circolone et al., 2012). Alternatively, information about transition between the different structural states and allosteric information flow can be obtained by molecular dynamics (MD) simulations (Karplus and McCammon, 2002). In recent years a number of MD simulations have been conducted on various LOV domains and full-length LOV photoreceptors (Dittrich et al., 2005; Freddolino et al., 2006; Peter et al., 2010, 2012a,b; Song et al., 2011; Freddolino et al., 2013). However, the issue as to how photon absorption by the FMN molecule and subsequent adduct formation results in a signal relay to fused effector domains remains far from resolved. The common feature, that evolved from those simulations on LOV domain systems from different organisms, which is corroborated by experimental evidence, is the role of a highly conserved glutamine residue (Q513 in AsLOV2, Q123 in YtvA and YF1), whose conformation is directly linked to photoreceptor activation (Nash et al., 2008; Avila-Perez et al., 2009). The question thus arises whether the mode of information flow from the photon absorbing FMN molecule to structurally conserved N- and C-terminal helical extensions (A'α and Jα) and consequently to fused effector domains is at atomic level conserved between plant and bacterial LOV photoreceptors, especially as the latter ones have so far not been studied by MD methods, i.e., how do small-scale conformational changes in the vicinity of FMN chromophore impact on the conformation of N- and C-terminally located structural elements.

**Figure 1:**
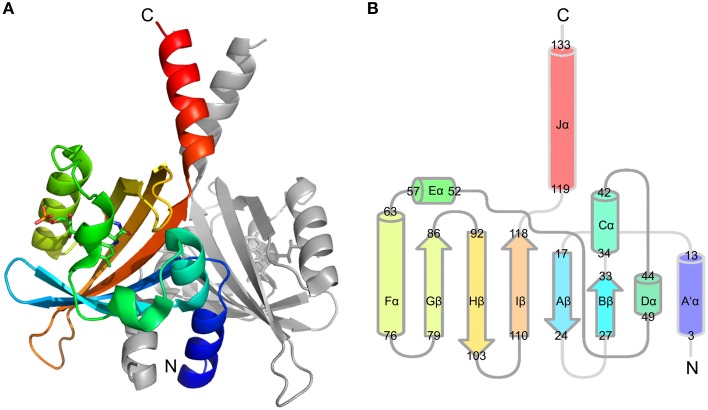
**PpSB1-LOV dimer structure (PDB-ID: 3SW1) (A) and conserved topology of LOV secondary structural elements (B)**. Chain A is colored using rainbow coloring from the N- to the C-terminus The FMN chromophore is shown in stick representation. **(B)** Depicts the LOV/PAS topology of PpSB1-LOV with α-helices shown as cylinders and β-sheets drawn as arrows. The non-canonical A'α- and Jα-helical extensions protrude from the LOV core. Numbers refer to amino acids at the boundaries of the respective secondary structural element.

We recently reported the X-ray crystal structure of a so-called short LOV protein PpSB1-LOV from *Pseudomonas putida*, obtained from crystals grown under constant low-light illumination (Figure 1A) (Circolone et al., 2012). Like other LOV domains, PpSB1-LOV possesses a typical mixed α/β Per-ARNT-SIM (PAS) fold in the topological order Aβ-Bβ-Cα-Dα-Eα-Gβ-Hβ-Iβ (Figure 1B). The FMN binding pocket is constituted by the β-scaffold surrounded by the three helices with the FMN molecule anchored above the central Iβ sheet. Outside of the canonical LOV-core domains the N-terminal A'α helix and the C-terminal Jα-helix protrude from the LOV core, largely constituting the LOV-LOV dimer interface (Figure 1). In contrast to fast-cycling phototropin LOV sensor domains, such as the LOV2 domain of *Avena sativa* phototropin 2 (Swartz et al., 2001), PpSB1-LOV possesses a very slow dark recovery with a long lived light state (light-state lifetime: approx. 2500 min; at 20°C) (Jentzsch et al., 2009). Unlike other LOV photoreceptors, PpSB1-LOV lacks a fused effector domain (Krauss et al., 2005; Jentzsch et al., 2009; Circolone et al., 2012; Rani et al., 2013). Similar architectures are found widespread throughout the bacterial world (Losi and Gärtner, 2008; Möglich et al., 2009; Rani et al., 2013), with short (effector-less) LOV proteins representing the third largest group of bacterial LOV photoreceptors (Losi and Gärtner, 2008). With respect to the observed dimeric arrangement, PpSB1-LOV strongly resembles the arrangement seen in the recently obtained dark-state structure of YF1 (Diensthuber et al., 2013) and probably YtvA (Ogata et al., 2009; Engelhard et al., 2013). Given this similar structural arrangement, i.e., parallel arrangement of the LOV-core domains in the homodimeric protein with protruding N- and C-terminal coaxial coiled-coil extensions, it is tempting to speculate that PpSB1-LOV and YtvA-LOV in YF1 undergo grossly similar light-dependent structural changes. Unfortunately, no dark-state crystal structure of PpSB1-LOV is yet available. Therefore, the structural basis of PpSB1-LOV photoactivation and intramolecular signal relay remains elusive. Moreover, with regard to the FMN-cysteinyl-thiol adduct the PpSB1-LOV light-state structure represents a “mixed state” as no clear evidence for the presence of the Cys53-SG FMN-C4a covalent linkage in the electron density map was observed (Circolone et al., 2012). Upon close inspection of the electron density around the FMN-C4a atom *sp3* hybridization of the 4a carbon atom can be inferred, which is in contrast to a planar conformation seen in LOV dark-state structures (Circolone et al., 2012). Moreover, in the PpSB1-LOV light-state structure, Q116 (corresponding to Q123 and Q513 of YtvA-LOV and AsLOV2) depicts two possible hydrogen bonds with the FMN isoalloxazine ring, namely FMN-O4 …NE2-Q116 (2.75 Å) and FMN-N5 …NE2-Q116 (2.87 Å); (Circolone et al., 2012). This is in contrast to previously reported photoexcited state structures of LOV domains, where a flipping of the Gln side chain oxygen (OE1) and amide (NE2) atoms was proposed as a consequence of illumination (Crosson and Moffat, 2002; Fedorov et al., 2003; Möglich and Moffat, 2007; Zoltowski et al., 2007; Vaidya et al., 2011). Please note, that none of the presently solved LOV X-ray structures does allow an unambiguous assignment of the respective side-chain atom positions solely based on electron density due to a too low resolution and high side chain disorder. Due to those structural features, it is currently not clear whether the reported PpSB1-LOV light-state structure correctly depicts all structural consequences of light-state formation. Therefore, since no PpSB1-LOV dark-state structure is available, no conclusion can be drawn about structural differences between the dark- and light state.

In order to address those open questions we performed molecular dynamics simulations on both the dark and light-adapted (adduct) state of PpSB1-LOV, by either introducing or omitting the FMN-C4a Cys53-SG adduct in the PpSB1-LOV (light-state) starting structure of the respective simulations. Based on those simulations we investigate the stability of the proposed PpSB1-LOV light-state X-ray structure and probe the conformational space the protein can sample after breaking the adduct, thus analyzing the early structural consequences of adduct breakage for PpSB1-LOV. Our simulations show that the presented PpSB1-LOV light-state X-ray structure remains globally stable during the simulation, i.e., with regard to the orientation of the C-terminal Jα coiled-coil interaction in the dimer, but reveals distinct conformational changes for side chains in the vicinity of the FMN chromophore. The direct comparison of light- and dark-state simulations, suggest different Jα-helix orientations as well as variable mobility between the two states. These changes coincide with a tilting of the Iβ and Hβ strands relative to the light-state starting structure and result in a correlated movement of both LOV core domains in the dimer. The observed global changes are most likely initiated by the reorientation of the conserved glutamine Q116 which flips its side chain oxygen atom position between Aβ (V19) in the dark state, and the Hβ strand (S98) in the light state, while enabling two potential hydrogen bonds to FMN-N5 and -O4, respectively. This local Q116 reorientation impacts on a salt-bridge network constituted by K117 and E96, which is stabilized in the light state, hence accounting for the observed decreased mobility. Based on these findings we propose a new signal-relay mechanism for dimeric LOV photoactivation and intramolecular signal-relay, assigning an alternative structural role for the conserved “flipping” glutamine. The proposed mechanism is discussed in light of universal applicability and its implications for the understanding and the design of LOV-based optogenetic tools.

## Materials and methods

### Simulation setup

The X-ray crystal structure of the short LOV protein PpSB1-LOV (Circolone et al., 2012) from *Pseudomonas putida* (PDB-ID: 3SW1) solved under constant illumination was used as basis for the modeling of the light- and dark-adapted state and subsequent molecular dynamics calculations using YASARA Structure (Krieger et al., 2004; Krieger and Vriend, 2014) (Ver. 14.7.17) software. The dark adapted structure was constructed by omitting the covalent bond between C53 and FMN. In the light-state starting structure the covalent bond between the C53-SG atom and the FMN-C4a atom was computationally introduced. The starting structures were protonated using the implemented pka-prediction and hydrogen bond network optimization algorithm (Krieger et al., 2012) and solvated in a periodic box (Krieger et al., 2006) using constrained TIP3P (Miyamoto and Kollman, 1992) water molecules. The box was neutralized at pH 7.4 using 0.9% NaCl solution and the water density was equilibrated to a final water density of 0.997 g/ml at 298 K. All simulations were performed utilizing the AMBER03 and the AMBER99 (see Supplementary Figure 8) (Wang et al., 2000; Duan et al., 2003; Krieger et al., 2004) force field for the protein residues and the general AMBER force field (Wang et al., 2004) (GAFF) using AM1-BCC (Jakalian et al., 2002) charges for the cofactor (parameters for the covalent Cys-FMN adduct are listed in Supplementary Table 2) and the default value for electrostatic cutoff (7.86 Å) was used with Particle Mesh Ewald algorithm (Essmann et al., 1995) for long range electrostatics utilizing 128 gridpoints on a 0.7 Å grid. The structure was initially minimized (Krieger et al., 2002) using first steepest descent without electrostatics to remove steric clashes and subsequently relaxed by steepest descent minimization and simulated annealing from 298 K (timestep 2 fs, atom velocities scaled down by 0.9 every 10th step) until convergence was reached, i.e., the energy improved by less than 0.05 kJ/mol per atom during 200 steps. Molecular dynamics calculations in an NPT ensemble using constrained bond length to all hydrogen atoms (Hess et al., 1997; Miyamoto and Kollman, 1992) were performed at 298 K and a solvent density of 0.997 g/ml using temperature rescaling the atom velocities using a modified Berendsen thermostat to slowly heat up the minimized system during an equilibration phase until the target temperature and density was reached. The simulation time step was 1.33 fs for intermolecular and 4 fs for intramolecular interactions to speed up the simulation and snapshots were saved every 25 ps. The MD-simulations were performed in three independent runs with different initial velocities for each system over 25, 45, and 95 ns and the trajectories were analyzed using YASARA (Krieger and Vriend, 2014) Structure and VMD (Humphrey et al., 1996).

### H-bonding and salt-bridge analysis

To obtain information about interactions in the vicinity of FMN chromophore distances and angles between relevant donor-acceptor pairs was obtained from the trajectories by using VMD. The corresponding distance and angle data was analyzed by using a custom Perl script employing moderate donor-acceptor distance (D-A) and angle (D-H ^…^ A) cutoff values of 3.2 Å and 130–180°, respectively (Jeffrey, 1997; Steiner, 2002). The script returns percent occupancy for the respective interaction for the trajectory. The corresponding values for all trajectories are listed in Supplementary Table 1. For the identification of salt-bridges the distance between the K117-NZ and E96-CD was measured and a distance cut-off of 5 Å was used for identification of a salt-bridge. This larger distance measure was chosen to avoid ambiguity due to E96-OE1/OE2 rotation (Barlow and Thornton, 1983; Xu et al., 1997).

### Jα-crossing angle analysis

The crossing angle of the Jα helices and A'α helices between the two subunits was calculated over the trajectories using YASARA by plotting a normal vector along the backbone atoms of the respective residues constituting the helix (Jα: residues 120–132; A'α: residues 3–13) and analysing the angle between the two normal vectors. The corresponding helix-crossing angle data was plotted as frequency distribution by using GraphPad Prism (GraphPad Software Inc. La Jolla, CA, USA). The average (mean) helix-crossing angles as well as the associated standard deviations were derived from the corresponding frequency distribution analyses.

### Dynamic cross-correlation analyses

Dynamic cross correlation analysis of all residue RMSD were performed using YASARA to deduce correlated movements of all residue pairs. The values in the DCCM range from −1 (perfectly anti-correlated) to +1 (perfectly correlated). The values along the diagonal are +1 (selfcorrelation). The DCC between a residue pair i and j is obtained with the following formula, where d is the displacement between the current position and the average position, and the angle brackets indicate the average over all sample snapshots.

$$\begin{array}{l} {DCCM}_{i,j}=\frac{\langle {\vec{d}}_{i}\cdot {\vec{d}}_{j}\rangle }{\sqrt{\langle {{\vec{d}}_{i}}^{2}\rangle \cdot \langle {{\vec{d}}_{j}}^{2}\rangle }} \end{array}$$

All corresponding dark- and light-state dynamic cross-correlation matrices were combined into average dark- and light-state matrices and a light-dark plot was generated by subtracting the averaged light- and dark-state matrices to identify changes in correlated motions between the two states. All matrix operations were carried out with Matlab R2014b (Mathworks GmbH, Ismaning, Germany).

## Results

The question as to how the light signal is relayed from the site of photon capture in the LOV domain active site to N- and C-terminal helical linker elements and consequently to fused effector domains in full-length oligomeric multi-domain LOV photoreceptors remains, despite extensive experimental efforts, still largely unresolved. This is due to the fact that all photoexcited state X-ray structures, i.e., for LOV proteins crystallized in the dark and illuminated immediately before data collection, show only small structural changes compared to the corresponding dark-state structures (Crosson and Moffat, 2002; Fedorov et al., 2003; Halavaty and Moffat, 2007; Möglich and Moffat, 2007; Zoltowski et al., 2007; Endres et al., 2015). Here, the crystal-lattice probably impedes larger scale structural changes. Moreover, no atomic resolution dark or light-state NMR structures, which would resolve this issue, have been reported for a LOV protein in solution. Thus, MD simulations represent the ideal technique to reveal possible structural consequences of photoactivation as the covalent adduct, i.e., the most salient feature of the LOV domain light state, can easily be introduced or omitted in the corresponding light- or dark-state simulations.

### The previously published PpSB1-LOV light-state structure remains globally stable over the simulation trajectory

Given the structural features of the recently solved PpSB1-LOV light-state X-ray structure, i.e., lack of clear electron density for the covalent FMN-cysteinyl-thiol adduct and the orientation Q116 side chain forming two possible hydrogen bonds with the FMN molecule, it is not clear if the structure depicts all structural features of a fully populated light state. To address this issue we computationally introduced a covalent linkage between the FMN-C4a atom and C53-SG atom. For simulation of the dark state, this covalent bond was omitted. We performed three independent simulations for the dimer of PpSB1-LOV in the dark state and three simulations for the light state (Table 1).

**Table 1:** **Summary of the performed simulations**.

**Name**	**State**	**Duration (ns)**	**RMSD (full)^$^(Å)**	**RMSD (core)^§^(Å)**	**RMSD (A'α)^§^(Å)**	**RMSD (Jα)^§^(Å)**
1D	Dark	25	1.84	2.87	2.60	2.31
2D	Dark	45	1.86	2.66	2.37	3.25
3D	Dark	95	1.82	2.42	2.17	2.70
Average	Dark		1.84	2.65	2.38	2.75
1L	Light	25	1.82	3.04	1.87	3.01
2L	Light	45	1.61	2.37	1.97	2.21
3L	Light	95	1.64	2.32	1.74	1.94
Average	Light		1.66	2.44	1.82	2.18

The trajectories were superimposed over the backbone atoms of both chains^$^ or over chain A^§^. RMSD values were calculated for the full length dimer (including Jα and A'α helix), the respective core domain (residues 17–117), the A'α helix (residues 3–13) and the Jα helix (residues 120–132) of chain B, relative to the starting structure. The reported average values were weighted for the duration of the respective trajectory.

**Figure 2:**
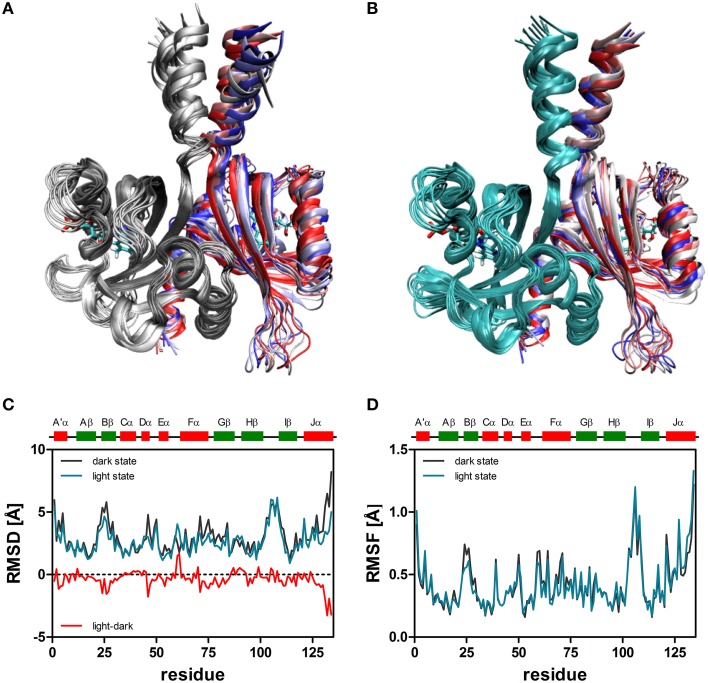
**Superimposition of 10 snapshots (Δt = 5 ns) of representative (2D, 2L) dark-state (A) and light-state trajectories (B)**. The snapshots were superimposed with VMD over all backbone atoms of chain A, colored in gray (dark-state) and cyan (light state). In both panels chain B is color coded by simulation time (*t* = 0: dark red; *t* = 45 ns: dark blue). The FMN cofactor is shown in stick representation in both subunits. **(C)** Per residue RMSD of the backbone atoms derived for chain B of the dark-state (dark gray) and the light-state (cyan) trajectories. The red line depicts light-dark RMSD values, with negative values indicating larger changes in the dark state. **(D)** Residue-resolved RMSF values for the dark-state (dark gray) and light-state (cyan) trajectories. Above the graph, LOV domain secondary structure elements are shown with α-helices in red and β-strands in green.

When the resulting trajectories are superimposed globally over the backbone atoms of the dimer, an average RMSD over the backbone atoms of 1.84 Å (dark) 1.66 Å (light) can be calculated, suggesting that larger structural changes do occur during the dark-state simulation compared to the light-state simulation. In order to better visualize potential inter-domain movements, the dark- and light-state trajectories were superimposed over all backbone atoms of chain A (Figures 2A,B). Potential domain rearrangements become then more apparent for chain B of the dimer. Especially, the N- and C-terminal A'α and Jα helices show increased RMSDs in the dark-state simulation. The corresponding RMSD values for the Jα helix (residues 120–132) are 2.75 Å (dark state) and 2.18 (light state). Likewise, for the A'α helix higher RMSD values are observed (dark state: 2.38, light state: 1.82). A residue-resolved RMSD plot (Figure 2C), as well as the corresponding heatmaps (Supplementary Figures 1, 2) pinpoint regions showing increased structural changes in the dark-state (Figure 2C; negative values in the light-dark plot). Those regions are (i) the N-terminal A'α helix, (ii) the Aβ-Bβ loop, (iii) parts of the Fα helix and the adjacent Gβ strand as well as the C-terminal Jα-helix. In the corresponding RMSF plot (Figure 2D) the N- and C-terminal A'α and Jα helices, Aβ-Bβ loop as well as the Hβ-Iβ loop show increased fluctuations. The Hβ-Iβ loop shows similar fluctuations in both the dark- and light-state simulations (Figure 2D).

### Local structural changes in the PpSB1-LOV active site induced by adduct formation

**Figure 3:**
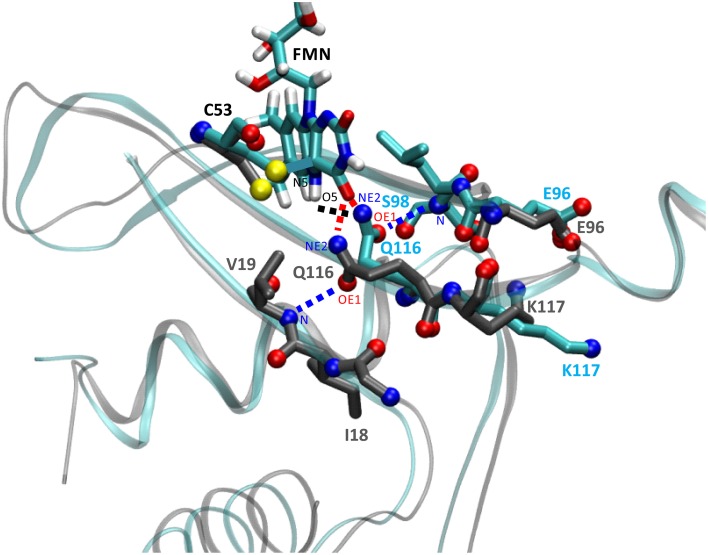
**Illustration of the structural differences between dark- and light-state simulations in the vicinity of the FMN chromophore**. The figure shows a representative state (averaged over 100 simulation frames) at a later time of the dark-state and light-state trajectory (2D, 2L). Relevant side chains are shown in stick representation with side chain nitrogen and oxygen atoms as CPK spheres. Residue carbon atoms are color-coded representing the dark-state (carbon atoms in dark gray) and light-state (carbon atoms in cyan). Potential H-bonds or electrostatic interactions are depicted as dashed lines (for details see the main text). The light-state Q116-NE2 … FMN-N5-H5 H-bond is shown in black, the dark- and light-state Q116-NE2 … FMN-O4 H-bonds are depicted in red and the Q116-OE1 … V19-N interaction (dark-state) or the Q116-OE1 … S98-N H-bond (light state) is drawn in blue. For orientation and clarity the photoactive cysteine (C53), the FMN chromophore (in the light state) as well as parts of the protein backbone are shown in gray (dark state) and cyan (light state). Besides Q116, the side chains of K117 (Iβ) and E96 (Hβ) experience a correlated reorientation (for details see the main text).

In order to address details of the structural changes that occur between the transition from dark- to light state we superimposed both trajectories over all backbone atoms of chain A. Potential consequences of photoactivation were analyzed for residues in the immediate vicinity of the FMN chromophore, i.e., the conserved glutamine Q116 as well as neighboring residues on the Aβ, Hβ, and Iβ strand (Figure 3). In the PpSB1-LOV light-state structure (PDB-ID: 3SW1) the Q116 side chain orientation facilitates two hydrogen bonds to the FMN chromophore (Q116-NE2 … FMN-N5, 2.87 Å; Q116-NE2 … FMN-O4, 2.75 Å). The Q116 side chain oxygen atom (OE1) faces toward the Aβ strand with a distance of 3.84 Å to the backbone amide of V19. To obtain information about the H-bonding interactions in the FMN binding pocket we analyzed all trajectories for the above outlined interactions (Supplementary Table 1). As criteria for the presence of an H-bond we used moderate cut-off values for distance (D…A; 3.2 Å) and angle (D-H…A; 130–180°) (Jeffrey, 1997; Steiner, 2002). For all trajectories H-bond occupancy was calculated as described in the Materials and Methods Section.

**Figure 4:**
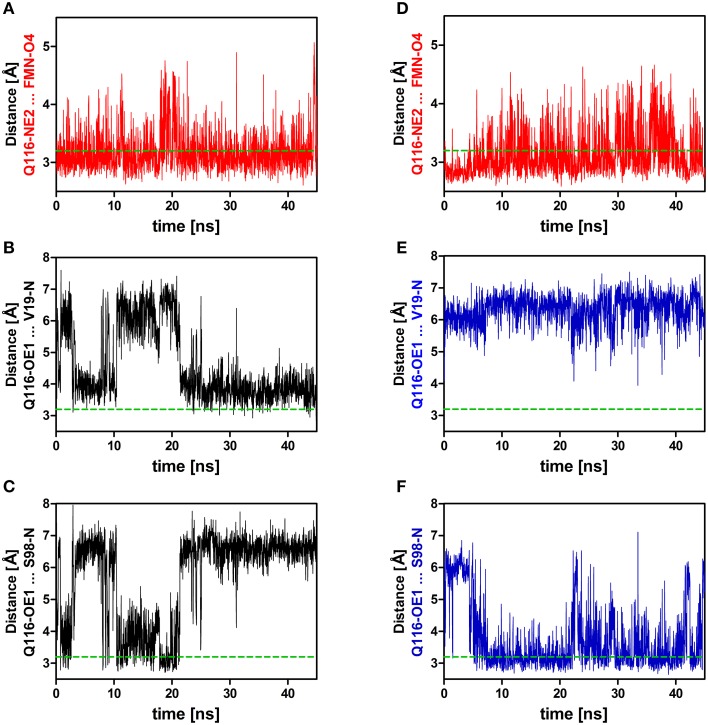
**Relevant distances between the side chain nitrogen (NE2) and oxygen (OE1) atoms of Q116 and the FMN chromophore (A,D) as well as to the backbone amide atoms of V19 and S98 (B,C,E,F)**. On the left side **(A–C)** distances derived from the dark-state trajectory are shown, while on the right side **(D–F)** distances derived from the light-state trajectory are depicted. The dashed green line represents the 3.2 Å hydrogen bonding distance cut-off (Jeffrey, 1997; Steiner, 2002).

Applying those criteria, in the dark state (1D, 2D, 3D), only the H-bond between Q116-NE2 and the FMN-O4 atom (via 2HE) is retained (present in about 49% of the trajectory time steps), while no H-bond is formed to FMN-N5 (occupancy below 5%) (Supplementary Table 1; Figures 3, 4A; Supplementary Figures 3, 4A). The Q116 side chain oxygen (OE1) is facing toward the backbone amide of V19 in the dark state, with an average distance above the 3.2 Å cut-off set for a hydrogen bond, but nevertheless establishing a weak electrostatic interaction (Figure 4B, Supplementary Figures 3, 4B). The corresponding interaction is absent in all the light-state trajectories (Figures 3, 4E; Supplementary Figures 3, 4E).

In the light-state trajectories (1L, 2L, 3L) the FMN-Q116 the H-bond between the Q116-NE2 atom and the FMN-O4 (via 2HE and 1HE) is retained (present in about 39 and 9% of all trajectory time steps; Supplementary Table 1; Figures 3, 4D, Supplementary Figures 3, 4D). Additionally H-bonding interactions are possible between Q116-NE2 and the FMN-N5 atom (via the newly protonated FMN-N5 H5-atom) (present in about 9% of all trajectory time steps; Supplementary Table 1, Figure 3). At the same time the FMN-OE1 atom flips toward the backbone amide of S98 on Hβ in the light state, establishing a new electrostatic interaction (Figures 3, 4F; Supplementary Figures 3, 4F). Applying the above described H-bonding selection criteria an H-bond is detected in about 23% of all trajectory time steps (Supplementary Table 1). In the dark state, this conformation is only present in about 10% of all trajectory time steps (Supplementary Table 1). Thus, while the overall dimer arrangement seen in the PpSB1-LOV light-state X-ray structure remained stable over the light-state simulation time (Figure 2) pronounced side chain rearrangements in the vicinity of the FMN chromophore occur, corroborating the “mixed” state nature of the recently solved PpSB1-LOV X-ray structure.

### Adduct formation induces the displacement of the Hβ and Iβ strands impacting an inter-subunit salt-bridge network

In the PpSB1-LOV light-state X-ray structure, the LOV-LOV dimer interface is largely constituted, by hydrophobic interactions of the A'α helices, the Jα-helices and interfacial residues of the Hβ and Iβ strands (Circolone et al., 2012).

**Figure 5:**
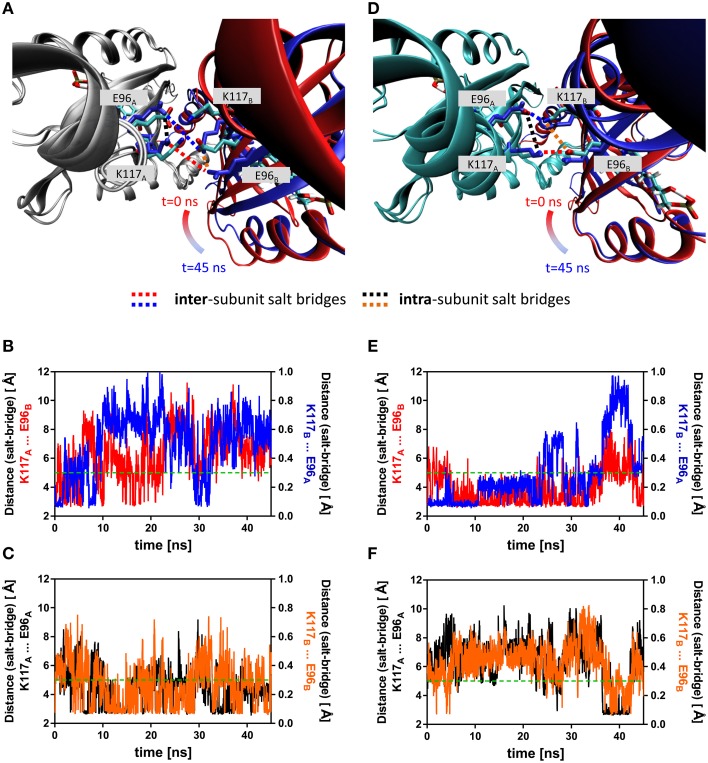
**Overlay of early and late representative snapshots from of the dark- and light-state trajectories (A,D)**. The presented snapshots were averaged over 100 frames to illustrate a representative state rather than single snapshot. The snapshots of representative simulations (2D, 2L) were superimposed over chain A of the dimer, colored in gray (dark state) and cyan (light-state). Chain B is shown in cartoon representation with the snapshots color-coded according to simulation time (*t* = 0 ns; red, *t* = 45 ns; blue). The K117 and E96 side chains, which can form an intra-subunit salt-bridge in the dark- and light state and an inter-subunit salt-bridge in the light-state are shown in stick representation with early snapshot (*t* = 0 ns) colored according to element (nitrogen in blue, oxygen in red and carbon in cyan). The K117 and E96 side chains of the late snapshot are shown in blue to illustrate the observed structural change. Inter-subunit salt-bridges are highlighted by blue and red dashed lines and intra-subunit salt-bridges by black and orange dashed lines. **(B)** Distance between atoms that would constitute the inter-subunit K117-*E96*' salt-bridge (labeled in **(A)** with red and blue dashed lines) in the dark state. **(C)** Distance between atoms constituting the intra-subunit K117-E96 salt-bridge (labeled in **(A)** with black and orange dashed lines) in the dark state. **(E)** Distance between atoms that constitute the inter-subunit K117-*E96*' salt-bridge (labeled in **(D)** with red and blue dashed lines) in the light state. **(F)** Distance between atoms that constitute the intra-subunit K117-E96 salt-bridge (labeled in **(A)** with black and orange dashed lines) in the light state. The dashed green line represents the 5 Å distance cut-off used for assignment of a salt-bridge (see Materials and Methods Section for details).

To illustrate the observed effects, an early representative state (averaged over the first 100 frames) and a late representative state (averaged over the last 100 frames) of the dark state (2D) trajectory is shown (Figure 5A). Here, the trajectory snapshots were superimposed over the backbone of chain A and chain B was colored according to simulation time. A displacement of the whole domain is visible from *t* = 0 (chain B colored in red) to *t* = 45 ns (chain B colored in blue). A similar overlay of early and late frames of the light-state trajectory did not reveal a similar subunit reorientation (Figure 5D). Over the trajectories this trend in displacement can be quantified e.g., for the Hβ and Iβ strands (RMSD Hβ (residues 94–104): dark state: 1.11 Å, light state: 0.99 Å; RMSD Iβ (residues 109–117): dark state: 0.93 Å, light state: 0.75 Å). Additionally, tilting of the C-terminal end of the Iβ strand toward the dimer interface and hence away from the respective core domain can be observed in the light-state simulations relative to the dark-state simulations (Supplementary Figure 9).

Additionally, a salt-bridge network between Hβ and Iβ can be identified in the PpSB1-LOV light-state X-ray structure which is constituted by the residues K117 and E96 of opposing subunits (Figures 5A,D). In both the dark- (Figure 5A) and light-state simulation (Figure 5D) different E96-K117 salt-bridge arrangements are observed. For all trajectories salt-bridge occupancy was calculated as described in the Materials and Methods Section. In both states E96 and K117 can form both an inter-subunit salt-bridge as well as an intra-subunit salt-bridge. In all dark-state simulations at least one inter-subunit salt-bridge is present, but the occupancy is on average below 50% (Figure 5B; Supplementary Figures 5, 6). In all three light-state simulations the occupancy for the same inter-subunit salt-bridge is increased to above 75% (Figure 5E, Supplementary Figures 5, 6). In all light- and dark-state simulations an intra-subunit salt-bridge between the same residues can be formed (if one of the inter-subunit salt-bridges is absent) (Figures 5C,F; Supplementary Figures 5, 6). This switching behavior suggests, that adduct formation may shift a pre-existing equilibrium toward the stabilization of the inter-subunit salt-bridge network.

### The orientation of protruding Jα and A'α helices is influenced by adduct formation

**Figure 6:**
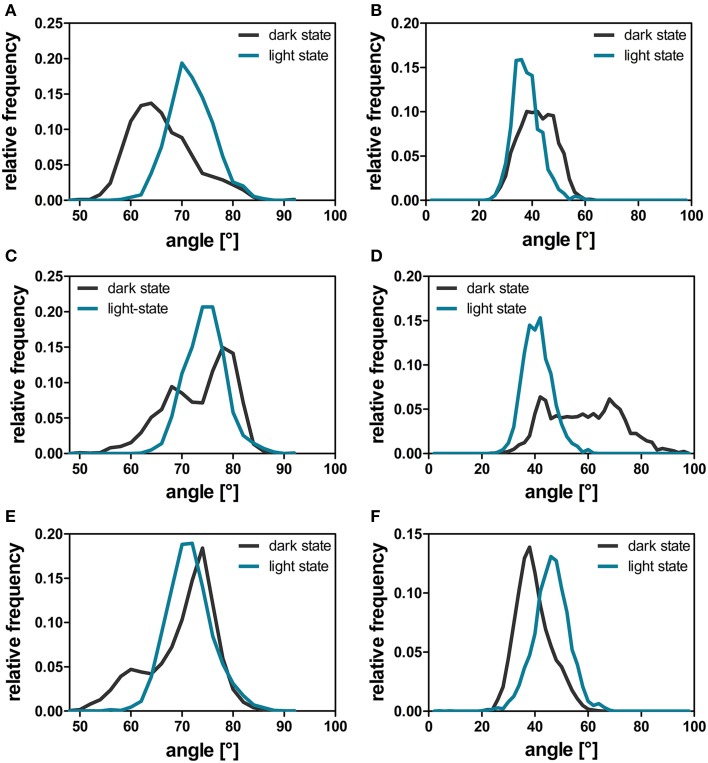
**A'α and Jα-crossing angles over the dark-state (dark gray line) and light-state (cyan line) trajectories**. The helix angle between the A'α **(A,C,E)** and Jα helices **(B,D,F)** between chain A and chain B was calculated with YASARA for each time step by plotting a normal vector along the backbone atoms of the respective residues constituting the helix (A'α: residues 119–133; Jα: residues 3–13). The respective angles were analyzed for all dark- and light-state trajectories as described in the Materials and Methods Section. The panels depict the data derived from the three different simulation runs: 1D, 1L **(A,B)**; 2D, 2L **(C,D)**, and 3D, 3L **(E,F)**.

The comparison of light- and dark-state simulations reveals different Jα-helix orientations as well as variable Jα mobility between the two states in both the short and the longer trajectories (Figure 6). The depicted helix crossing-angle distribution plots were obtained from the crossing angle of the normal vector as described in the Materials and Methods Section. The light-state conformation seems to be stable with average crossing angles of 38° ± 5° (1L), 41° ± 5° (2L), and 46° ± 6° (3L), respectively (Figures 6B,D,F; cyan line). The corresponding dark-state simulation reveals an increased overall Jα conformational mobility sampling multiple helix angles between 24° and 96° degree over the short trajectories (1D, 2D) (Figures 6B,D, dark gray line). In the longer trajectory a sharper angle distribution, with an average value of 39° ± 6°, is found for the dark-state trajectory (Figure 6F).

For the A'α helix similar angle distributions are observed for all trajectories revealing a broader angle distribution between 50° and 90° in the respective dark-state simulations (Figures 6A,C,E; dark gray line) and a sharper angle distribution in the light state (Figures 6A,C,E; cyan line).

### Correlated motions

**Figure 7:**
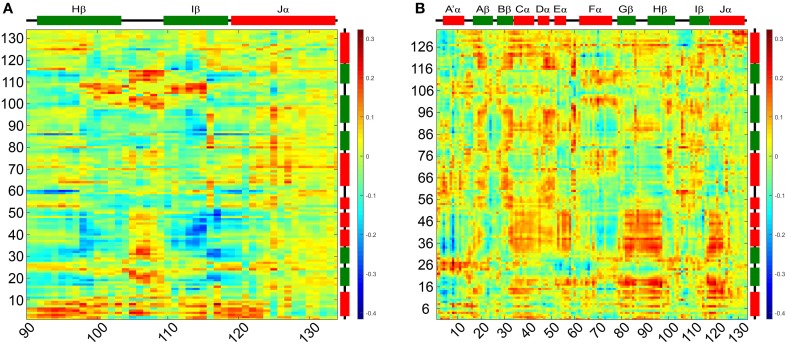
**Light-dark dynamic cross-correlation matrices (DCCMs) illustrating differences in pairwise correlated motions**. **(A)** Differences in cross correlations between residues 90–134 on the Hβ, Iβ, and Jα and chain A. **(B)** Differences in cross correlation between chain A and chain B of the dimer. Positive values (red) can result from two scenarios: (i) increase in residue pair correlation in the light state and (ii) increased anti-correlated motions in the dark state. All correlations discussed in the manuscripts are of the first category.

To evaluate correlation of the above outlined intra- and inter- subunit interactions and characterize structural differences between the two states we analyzed the correlated motions of all residue pairs along the dark- and light state trajectories. Dynamic cross-correlation matrices (DCCMs) were calculated as described in the Materials and Methods Section. The obtained dark- and light state DCCMs show typical intra-subunit correlation patterns, e.g., blocks along the main diagonal for consecutive helical motifs and lines perpendicular to the main diagonal indicating correlated motions of neighboring β-strands (Supplementary Figures 7A,C). To better characterize the differences in correlated motions between the light- and dark-state simulations we generated a light-dark difference DCCM (Supplementary Figure 7B). No major differences between the dark- and light-state correlations along the main diagonal are observed indicating no major structural rearrangements within the monomer. In Figure 7A the light-dark differences in cross correlations between residues 90–134 on the Hβ, Iβ, and Jα (X-Axis) and chain A (Y-Axis) are highlighted, showing increased correlated motions along the main diagonal for the Hβ-Iβ loop region (residues 100–110) in the light state. Additionally changes off the diagonal, indicating long-range interactions, are observed for Hβ/Iβ and Jα/A'α, visible as yellow region in the upper left corner of Figure 7A and red region in the lower part of Figure 7A. The residues E96, S98, Q116, and K117 are all showing increased correlated motions in the light state, within the monomer. In Figure 7B the light-dark differences in cross-correlation between chain A (X-axis) and chain B (Y-axis) are shown. Surprisingly, the differences between dark- and light state become more evident compared to the intramolecular correlations (Supplementary Figure 7B). This can be seen for example in the region of Gβ-Hβ (chain A) which shows a change in correlated motions together with the N-terminal region from A'α to Eα on chain B. The largest increase in correlated motions is observed between the Jα helices of chain A and B (Figure 7B; Supplementary Figures 7A,C).

## Discussion

### Photoactivation and signal-relay mechanism of PpSB1-LOV inferred from MD simulations

**Figure 8:**
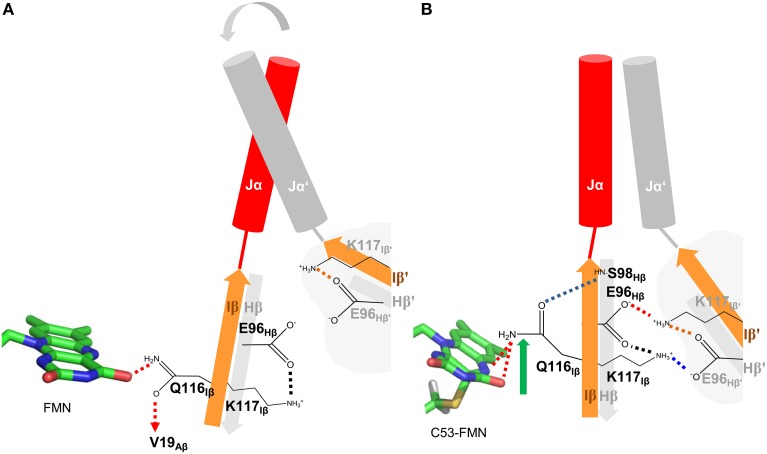
**Illustration of the signal relay mechanism proposed for PpSB1-LOV**. The Hβ (gray) and Iβ (orange) strands of both subunits, harboring the here identified mechanistically relevant amino acid residues (Q116, E96, S98) are shown. In the dark state **(A)**, Q116 is facing toward V19 on Aβ (indicated by a dashed arrow), while enabling one H-bond to the FMN molecule (red dashed lines). The respective Hβ-Iβ orientation facilitates the destabilization of an inter-subunit K117-*E96*′ salt-bridge which shows increased occupancy in all light-state simulations (red and blue dashed lines in **B**) resulting in meta-stable subunit interactions and increased Jα mobility in the dark state. In the light state **(B)**, Q116 flips toward S98 on Hβ (flipping indicated by green arrow) establishing a new H-bond between Q116-OE1 and S98-NH (blue dashed line). This results in a correlated displacement of the Hβ and Iβ strands, stabilizing the inter-subunit salt-bridge network (red and blue dashed lines) between K117 and E96 of opposing subunits. This stabilizing interaction “locks” the two subunits (including Jα and A'α) in an orientation as observed in the light-state X-ray structure.

Based on the presented simulations, the following photoactivation and signal relay mechanism can be inferred (illustrated in Figure 8). The recently solved crystal structure of PpSB1-LOV, obtained under illumination, can be assigned according to our simulations with high confidence to a metastable light-state. In the light state FMN-N5 protonation enables two potential H-bonds between the FMN molecule and Q116 (FMN-N5^…^Q116-NE2 (with the FMN-N5-H5 being the H-bond donor) and FMN-O4^…^Q116-NE2 (with Q116-NE2-2HE/1HE being the H-bond donor) (See Local Structural Changes in the PpSB1-LOV Active site Induced by Adduct Formation; Supplementary Table 1; Figure 8B). In contrast to the crystal structure a rotation of the Q116 side chain is observed. The Q116-OE1 atom flips toward S98 on the Hβ strand (compare Figures 8A,B; Q116 flipping indicated by green arrow). The Q116 side chain oxygen atom (OE1) is hereby most of the time oriented toward the S98 backbone amide (Figure 4F), thus “locking” the Q116 side chain at the back-face of the FMN molecule (Figure 8B).

This structural signal could be relayed via the Iβ backbone to K117 and via the Hβ backbone (Fenwick et al., 2014) to E96 as indicated by DCCM analyses (Figure 7A). As consequence, tilting of the C-terminal end of the Iβ strand (Supplementary Figure 9) toward the dimer interface influences the conformation of K117 which together with E96 on Hβ reorient to form an inter-subunit salt-bridge in the light state (See Adduct Formation Induces the Displacement of the Hβ and Iβ Strands Impacting an Inter-subunitsalt-bridge Network, Figure 5). This likely “locks” the two LOV subunits in a stable relative position as observed in the PpSB1-LOV light-state X-ray structure, with lower average RMSD values for the core domain, A'α and Jα (Table 1). This is also reflected in increased inter-subunit correlated motions in the light state (Figure 7B).

In contrast, in the dark state (Figure 8A), the Q116 side chain is oriented toward the FMN molecule, with one potential H-bond being formed between the Q116-NE2 atom and the FMN-O4 atom (see Local Structural Changes in the PpSB1-LOV Active Site Induced by Adduct Formation, Supplementary Table 1). The Q116 side chain oxygen (OE1) is mainly oriented toward V19 (Figure 4B) on the Aβ strand (Figure 8A). Due to this reorientation of Q116 toward Aβ compared to the light state (Q116 oriented toward S98 on Hβ), the stabilizing inter-subunit K117…*E96*′ – E96…*K117*′ salt-bridge breaks, enabling an increased mobility in the relative orientation of the two subunits (Figure 5A). This is reflected by higher average RMSD values for the core domain, A'α and Jα (Table 1). In addition, all light-state simulations reveal relatively narrow Jα and A'α crossing angle distributions, while multiple conformations are sampled in the corresponding dark-state simulations, indicating, that no stable dark-state equilibrium is reached on the employed simulation time scales. This is not surprising, since the experimentally determined light-state lifetime of PpSB1-LOV is with about 2500 min (Jentzsch et al., 2009) at 20°C far outside of achievable simulation time scales. Therefore, we cannot conclude on the equilibrium structure of the PpSB1-LOV dark state, as conformational changes which might not be observed on our simulation timescale, i.e., further subunit rotation, increased A'α displacement and or subunit dissociation could occur. Those issues, as well as the feasibility of our mechanistic proposal, can only be unambiguously resolved if a dark-state X-ray structure or NMR data for the PpSB1-LOV protein becomes available.

The here proposed mechanism is globally similar to previous hypotheses, i.e., brought forward based on the dark-state X-ray structure of YF1 (Diensthuber et al., 2013), a recently engineered artificial photoreceptor containing the YtvA-LOV domain as sensory module (Möglich et al., 2009). This structure suggested decreased helix-crossing angles for the A'α-helix (29°) and Jα-helix (33°) in the dark state compared to the light-adapted state, which was modeled based on the PpSB1-LOV X-ray structure (A'α-helix: 70°; Jα-helix: 49°) (Diensthuber et al., 2013), which is corroborated by our simulations (See The Orientation of Protruding Jα and A'α Helices is Influenced by Adduct Formation, Figure 6F). NMR relaxation data for full-length YtvA in the dark state revealed a low order parameter (*S*^2^); (calculated from relaxation rates and heteronuclear NOEs) for the Jα-helix indicating a high degree of mobility (Jurk et al., 2011). This effect can also be observed in our simulations (see The Orientation of Protruding Jα and A'α Helices is Influenced by Adduct Formation, Figures 6B,D). Without either a dark state structure of PpSB1-LOV, a light-state structure of YF1 or full-length dark and light-state structures of YtvA it is impossible to delineate between the two scenarios.

For monomeric AsLOV2 Freddolino et al. (2013) recently presented a mechanistic proposal based on MD simulations, as to how photon capture by the FMN chromophore initiates the displacement of the Jα helix. The authors suggested that structural changes to the Iβ strand and the Hβ-Iβ loop lead to a tilting of the Iβ strand that forces a corresponding movement of the Jα helix, eventually resulting in a disruption of the native interface between the respective secondary structure elements. Those structural changes are globally similar to the structural changes which we suggest to occur in photoactivation of dimeric PpSB1-LOV, although in our case occurring in the dark-state simulation and not the light-state trajectory as observed by Freddolino and co-workers. However, this discrepancy and the apparent reversal of the reaction in PpSB1-LOV compared to AsLOV2 is most likely related to the fact that the here presented simulations start from a light-state structure obtained from crystals grown under constant illumination. In contrast, the AsLOV2 light-state structure is obtained by illuminating a dark grown crystal and hence larger-scale structural changes are most probably impeded by the crystal lattice. Thus, globally, the AsLOV2 light-state structure resembles the dark state. This assumption is corroborated by a small all-atom RMSD value of 0.608 Å between the dark- and light-state AsLOV2 X-ray structures. It is thus not surprising, that larger structural perturbations are observed in the dark-state simulation of dimeric PpSB1-LOV. Freddolino co-workers and Peter et al. (2010) noted that the Q513 sidechain samples several conformations in the light state. However, no correlation was found between the Q513 rotamerization state and larger-scale changes in the LOV domain structure except in cases where the Q513-FMN interaction was lost. It thus seems that in both PpSB1-LOV and AsLOV2 Q116/Q513 rearrangement is the trigger to initiate a structural relay mechanism most likely via Hβ/Iβ displacement/tilting. For monomeric AsLOV2 this could result in the dissociation of the C-terminal Jα helix from the core. For dimeric PpSB1-LOV increased inter-domain correlations (Figure 7B), strengthened inter-domain salt-bridge interactions (Figure 5) as well as a more defined A'α and Jα angle distribution (Figure 6) are observed, hinting at an essential functional role for residues in the dimer interface, which is constituted by Hβ/Iβ and the two terminal α-helices.

### Evidence for the conservation of the identified signaling mechanism in other LOV systems

We here identified a possible alternative mode of signal-relay in PpSB1-LOV from the site of photon capture, involving a conserved glutamine in the LOV domain FMN-binding site, to the N- and C-terminal helical extensions (A'α-helix and Jα-helix) outside the canonical LOV-core domain. In the corresponding light-state trajectory, Q116-OE1 reorients toward the backbone amide of S98, which is accompanied by a movement of the Iβ-strand and consequently a movement of the K117 toward *E96*′ (of the opposite subunit) hence stabilizing an inter-subunit salt-bridge, which connects the two LOV domains of the dimer, locking the dimer arrangement as well as the Jα and A'α interactions. As outlined above, the proposed photoswitching mechanism, is globally similar to the one proposed previously based on simulations of other LOV domains (Neiss and Saalfrank, 2004; Peter et al., 2012a,b; Freddolino et al., 2013) using a monomeric model setup, but differs with regard to certain aspects, i.e., the nature of the Q513 displacement in the light-state simulations and the impact of those changes on the structural regions neighboring the displaced/tilted Iβ strand.

The question thus immediately arises if this mode of signaling could be conserved in other LOV sensory systems, which possess similar structural elements. If the outlined signal-relay mechanism of PpSB1-LOV would be conserved in other LOV systems, one would expect to identify conserved residues at the respective key positions or at least find functional mutation data for the respective regions that would support their importance for signaling.

**Figure 9:**
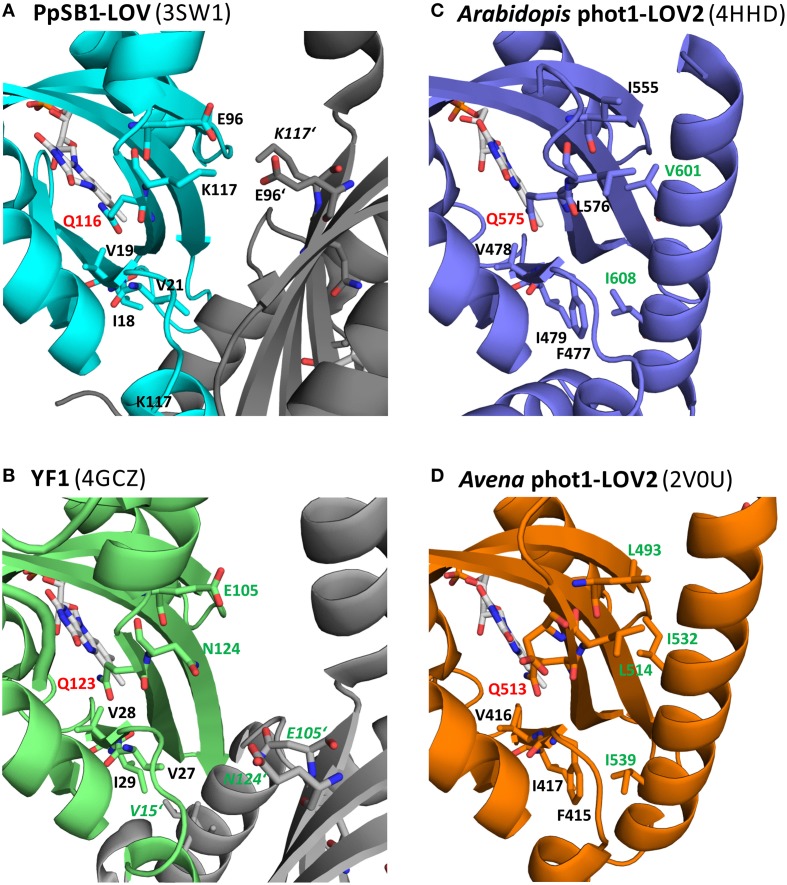
**Amino acid positions in three different LOV systems corresponding to the here identified key amino acids of PpSB1-LOV**. The conserved “flipping” glutamine is highlighted in red (Q116 PpSB1-LOV; Q123 YF1; Q575: At-phot1-LOV2; Q513: As-phot1-LOV2). The residues corresponding to the PpSB1-LOV E96-K117 salt-bridge network **(A)** are E105, N124 (YF1; **B**), I555, L576 (At-phot1-LOV2; **C**) and L514, L493 (As-phot1-LOV2; **D**). The position of V19, whose backbone amide group forms a weak electrostatic interaction with Q116-OE1 in the dark-state trajectories of PpSB1-LOV corresponds to V28 (YF1), V478 (At-phot1-LOV), and V416 (As-phot1-LOV2). Additionally neighboring residues, which protrude into the LOV-core / A'α interface, are shown in stick representation. Residues which upon mutation showed an altered signaling phenotype are highlighted in green.

Figure 9 shows a close-up view of the LOV domain active site of different LOV sensory systems, with the residues corresponding to the key residues identified for the PpSB1-LOV signal relay shown in stick representation. Additionally, residues, for which functional mutation data is available, displaying an altered signaling phenotype (either observed for the full-length photoreceptor in the biological context Harper et al., 2004; Jones et al., 2007; Avila-Perez et al., 2009; Gleichmann et al., 2013, or *in vitro* for the isolated LOV domain by biophysical means, Zayner et al., 2012), are depicted in stick representation and are labeled in green. In Figure 9A, the FMN binding site of PpSB1-LOV along with the here identified key amino acids in the vicinity of the chromophore, is shown. Additionally, the K117-E96 salt-bridge network which stabilizes the LOV-LOV interaction in the light-state is highlighted. In YF1 (Figure 9B) the position of K117 is occupied by asparagine (N124) and E96 is conserved (E105 in YF1). In the YF1 dimer structure E105 and N124 do not form a hydrogen-bond. Please note, that YF1 was crystallized in the dark, hence according to our here described model, a potential N124-E105 inter-subunit interaction would be expected to be broken. Given the overall structural similarity between PpSB1-LOV, YtvA, and YF1, it is tempting to speculate that in YF1 and YtvA, like in PpSB1-LOV a light triggered rotation of the two subunits relative to each other results in altered Jα and A'α interactions which are stabilized by an interaction between E105 and N124 of opposing subunits. *In vitro* and *in vivo* functional data for YtvA (Avila-Perez et al., 2009) and YF1 (Gleichmann et al., 2013) highlight the importance of both residues for the signal-relay since their mutation results in an altered signaling behavior. In analogy, Figures 9C,D depict the LOV domain active site of the phototropin 1 LOV2 domain of *Arabidopsis thaliana* (Figure 9C) and *Avena sativa* (Figure 9D). Like for PpSB1-LOV and YF1, the key residues for the proposed signal-relay mechanism are shown in stick representation. The positions of K117 and E96 of PpSB1-LOV are occupied by hydrophobic residues in AsLOV2 (L493 and L514) and AtLOV2 (I555 and L576). Those hydrophobic residues seem to “lock” the Jα-helix in place as observed in the respective dark-state X-ray structures. In light of the proposed signaling mechanism, reorientation of the active-site glutamine (AsLOV2: Q513; AtLOV2: Q575) would result in concomitant displacement of neighboring L515 (AsLOV2) or L576 (AtLOV2) which could trigger the release of the Jα-helix from the LOV core. Moreover, mutation of some of those residues (e.g., L493 and L514 in AsLOV2) resulted to a certain degree in altered structural changes *in vitro* (Harper et al., 2004; Jones et al., 2007; Zayner et al., 2013). While the here described hypothesis is certainly not mutually exclusive, as the respective mutational data can also be explained by other mechanistic proposals, such as the one brought forward for monomeric phototropin LOV sensor domains by Freddolino et al. (2013); (Outlined in Photoactivation and Signal-relay Mechanism of PpSB1-LOV Inferred from MD Simulations), it nevertheless provides an alternative scenario which could account for the observed signal relay especially for dimeric LOV photoreceptors. Further studies of site-directed mutants, both *in vitro* and in the biological context are needed to unequivocally delineate between the hypotheses described to date.

## Conclusions

Taken together, our simulations stress the importance of the conserved LOV domain glutamine for the overall signaling process and identify new key residues which might be involved in the signal relay from the site of photon capture to N- and C-terminally located effector domains via the A'α and Jα helical connectors. As outlined above, in light of functional mutation data, the proposed signal-relay mechanism might be universally applicable, but certainly not mutually exclusive, in the explanation of the signaling behavior of bacterial short LOV proteins, YtvA and homologous proteins as well as for the phototropin LOV system.

The proposed mechanism is elegant in two ways. First of all, it can explain available functional mutation data and, secondly, accounts for the observation that functionally dissimilar mutations can be introduced at presumed key positions without completely abolishing LOV functionality in terms of photocycling and conformational changes. From mutational studies of AsLOV2 it became apparent, that the only residue required for photocycling is the conserved photoactive cysteine and that many mutations even though intended to be disruptive only attenuated or even increased the light-dependent conformational change, i.e., Jα displacement and/or unfolding (Zayner et al., 2012; Zayner and Sosnick, 2014). In light of our mechanistic proposal, this can be explained, because the major atom partners involved the primary conformational switch, apart from the conserved glutamine, are backbone amide positions on Hβ and Aβ, which cannot be altered by mutation. Likewise the structural signal is proposed to be propagated *via* a displacement of the protein backbone from e.g., Q116 to K117 and S98 to E96 in PpSB1-LOV, which most probably cannot be directly influenced by mutation. Moreover, the proposed mechanism can account for the evolutionary plasticity of the LOV sensor domain, i.e., revealed by the multitude of different sensor-effector domain combinations in different LOV photoreceptors. According to our mechanistic proposal, the primary signaling event (glutamine displacement between Aβ and Hβ) can still be accommodated even if key functional amino acids (e.g., S98 and E96 on Hβ, K117 on Iβ and V19 on Aβ of PpSB1-LOV) are mutated, since the glutamine only switches the interaction between backbone amides of those residues. Hence mutation of those residues can in evolutionary terms be used to accommodate different interaction partners, i.e., the Jα-helix packing against the β-scaffold in the phototropin LOV system or LOV dimer stabilization via a salt-bridge (PpSB1-LOV) or hydrogen-bonding (YtvA, YF1) network.

At the same time, the presented mechanistic proposal has implications for the design and understanding of LOV-based optogenetic tools such as YF1 (based on YtvA) and those based on AsLOV2. If the general signal-relay mechanism is conserved between those systems, they represent an ideal testing ground for our proposal, as the here identified key residues can be easily mutated and screened for altered signaling phenotypes, which is not always so easy for the parent natural photoreceptor system. Currently, the design of LOV-based optogenetic switches is still a trial and error process, also due to the lack of an in-depth understanding of the mode of information flow from the site of photon capture to the fused effector domain *via* N- and C-terminal helical linker elements which proofed to be functionally important for the signal relay. Thus, the information gained in the here presented simulations can in the future contribute to the rational understanding and design of novel LOV-based optogenetic switches.

## Author contributions

US, KEJ, MB, and UK conceived the study. MB and UK designed the experiments. MB performed the simulations. MB and UK analyzed and interpreted the data. MB and UK drafted the manuscript. All authors critically revised and approved the final version of the manuscript.

### Conflict of interest statement

The authors declare that the research was conducted in the absence of any commercial or financial relationships that could be construed as a potential conflict of interest.
