# Supplementary material for: Light-induced structural changes in a short light, oxygen, voltage (LOV) protein revealed by molecular dynamics simulations—implications for the understanding of LOV photoactivation
Source: Front Mol Biosci. 2015 Oct 1;2:55. doi: 10.3389/fmolb.2015.00055 (PMC4589677; doi:10.3389/fmolb.2015.00055)
Supplement: Supplementary file 1 [file Presentation1.PDF]

Supplementary Material

Light-induced structural changes in a short light. oxygen. voltage (LOV) protein revealed by molecular dynamics simulations – implications for the understanding of optogenetic tools.

Marco Bocola[1*], Ulrich Schwaneberg[1], Karl-Erich Jaeger[2.3], Ulrich Krauss[2*]

[1]:Lehrstuhl für Biologietechnologie, RWTH Aachen University, Aachen. Germany
[2]:Institut für Molekulare Enzymtechnologie, Heinrich Heine University Düsseldorf, Forschungszentrum Jülich. Jülich. Germany
[3]:IBG-1. Biotechnology, Forschungszentrum Jülich. 52426 Jülich, Germany

*** Correspondence:**

Dr. Ulrich Krauss, Institut für Molekulare Enzymtechnologie, Heinrich Heine University Düsseldorf, Forschungszentrum Jülich, Wilhelm Johnen Strasse, D-52428 Jülich. Germany, u.krauss@fz-juelich.de

Dr. Marco Bocola, Lehrstuhl für Biologietechnologie, RWTH Aachen University, Worringerweg 3, D-52074 Aachen, Germany, m.bocola@biotec.rwth-aachen.de

1. Supplementary Tables

Supplementary Table 1: H-bonding analysis for selected residues in the FMN-binding pocket of PpSB1-LOV. The depicted percent occupancy values for the respective interaction were derived using a custom Perl script which analyses distance and angle data for the presence of an H-bond for every time step of the trajectory. Moderate distance (3.2 Å) and angle cut-off values (130 ° - 180 °) were employed for the calculation. Average values were weighted for the duration of the respective trajectory

			Q116-OE1-S98-N light		Q116-OE1-S98-N dark		Q116-OE1-V19-N light		Q116-OE1-V19-N dark	
**Run**	**force field**	**duration**	chain A	chain B	chain A	chain B	chain A	chain B	chain A	chain B
1	Amber ff03	45 ns	39.77	2.88	9.2	5.82	0	0	0.11	0
2	Amber ff03	25 ns	33.26	6.43	29.03	22.81	0	0	0	0.12
3	Amber ff03	95 ns	31.76	16.3	9.74	3.97	0	0	0.29	0.03
			mean		mean		mean		mean	
1	Amber ff03	45 ns	21.33		7.51		0.00		0.055	
2	Amber ff03	25 ns	19.85		25.92		0.00		0.06	
3	Amber ff03	95 ns	24.03		6.86		0.00		0.16	
**weighted average**			**22.66**		**9.92**		**0.00**		**0.12**	

			Q116-NE2-FMN-N5 light		Q116-NE2-FMN-N5 dark			
			via FMN-H5		via 1HE		via 2HE	
**Run**	**force field**	**duration**	chain A	chain B	chain A	chain B	chain A	chain B
1	Amber ff03	45 ns	1.74	24.85	3.1	5.93	3.38	7.32
2	Amber ff03	25 ns	6.88	3.22	1.5	2.53	10.52	8.41
3	Amber ff03	95 ns	8.91	5.6	4.16	0.11	2.12	1.72
			mean		mean		mean	
1	Amber ff03	45 ns	13.30		4.52		5.35	
2	Amber ff03	25 ns	5.05		2.02		9.47	
3	Amber ff03	95 ns	7.26		2.14		1.92	
**weighted average**			**8.57**		**2.77**		**4.00**	

Run	force field	duration	Q116-NE2-FMN-O4 light				Q116-NE2-FMN-O4 dark			
			via 1HE		via 2HE		via 1HE		via 2HE	
			chain A	chain B	chain A	chain B	chain A	chain B	chain A	chain B
1	Amber ff03	45 ns	15.57	0.98	49.97	70.59	0	0	55.65	54.99
2	Amber ff03	25 ns	1.01	0.11	55.13	75.65	0.12	0	36.29	52.53
3	Amber ff03	95 ns	7.78	13.48	30.76	11.99	0	0	53.67	38.52
			mean		mean		mean		mean	
1	Amber ff03	45 ns	8.28		60.28		0		55.32	
2	Amber ff03	25 ns	0.56		65.39		0.06		44.41	
3	Amber ff03	95 ns	10.63		21.38		0		46.10	
**weighted average**			**8.46**		**38.65**		**0.01**		**48.36**	

Supplementary Table 2: GAFF Parameter/Topology for the covalent cysteine-FMN adduct. The partial charges (last column) are calculated using AM1-BCC.

```
The following bond/angle/dihedral/improper parameters were either newly derived (because they are not part of the standard
AMBER03 force field) or modified:

S -c3                 1.821    225.800          Copied from ss-c3

o -c -ne            250.000    124.390          Force constant increased to avoid geometric distortions
n -c -ne             70.600    125.950          Copied from n -c2-n2
c -n -c             250.000    119.630          Force constant increased to avoid geometric distortions
S -c3-c              61.700    111.580          Copied from ss-c3-c
CT-S -c3             60.600     99.920          Copied from c3-ss-c3
S -c3-nh             67.100    107.445          Combined from ss-c3-ss and nh-c3-nh
c -c3-nh             65.800    113.910          Copied from c -c3-n3
nh-c2-ne            250.000    122.720          Force constant increased to avoid geometric distortions
c2-ne-c             250.000    118.530          Force constant increased to avoid geometric distortions
S -c3-c2             63.600    104.970          Copied from ss-c3-c2
c2-c3-c             250.000    109.730          Force constant increased to avoid geometric distortions
c2-c3-nh             66.500    111.470          Copied from c2-c3-n3
p5-os-c3            250.000    118.000          Force constant increased to avoid geometric distortions
o -p5-os            250.000    116.090          Force constant increased to avoid geometric distortions
o -p5-o             250.000    115.800          Force constant increased to avoid geometric distortions

X -S -c3-X     3      1.000      0.000     3    Copied from X -ss-c3-X

Topology for residue CYA A 53, net charge is 0.00
Created with YASARA AutoSMILES: www.yasara.org/autosmiles
CYA  INT    1
CORR OMIT DU   BEG
   0.00000
   1  DUMM  DU    M    0  -1  -2     0.000     0.000     0.000   0.00000
   2  DUMM  DU    M    1   0  -1     1.000     0.000     0.000   0.00000
   3  DUMM  DU    M    2   1   0     1.000    90.000     0.000   0.00000
   4  N     N     M    3   2   1     1.000    90.000    90.000  -0.43590
   5  H     H     E    4   3   2     1.011   149.520   -21.553   0.29010
   6  CA    CT    M    4   3   2     1.475    70.051  -134.386  -0.03050
   7  HA    H1    E    6   4   3     1.090   107.817    19.348   0.13210
   8  C     C     M    6   4   3     1.548   115.301   -99.117   0.62480
   9  CB    CT    3    6   4   3     1.538   109.146   136.778  -0.03300
  10  HB    H1    E    9   6   4     1.091   109.765   -47.410   0.07900
  11  HB    H1    E    9   6   4     1.089   107.534    69.786   0.07900
  12  O     O     E    8   6   4     1.237   119.530   158.135  -0.57320
  13  SG    S     S    9   6   4     1.826   112.816  -168.225  -0.13230

IMPROPER
 -M   CA   N    H
 CA   +M   C    O
```

```
Topology for residue FMN A 500, net charge is -2.00
Created with YASARA AutoSMILES: www.yasara.org/autosmiles
FMN  INT    1
CORR OMIT DU   BEG
   0.00000
   1  DUMM  DU    M    0  -1  -2     0.000     0.000     0.000   0.00000
   2  DUMM  DU    M    1   0  -1     1.000     0.000     0.000   0.00000
   3  DUMM  DU    M    2   1   0     1.000    90.000     0.000   0.00000
   4  N1    ne    S    3   2   1     1.000    90.000    90.000  -0.67642
   5  C2    c     B    4   3   2     1.391    48.924  -143.843   0.83504
   6  C10   c2    B    4   3   2     1.284    93.170   -17.476   0.68388
   7  C4A   c3    M    6   4   3     1.534   120.329   -30.274   0.18324
   8  N3    n     B    5   4   3     1.321   123.364    77.240  -0.60440
   9  N10   nh    B    6   4   3     1.374   120.864   151.169  -0.55442
  10  O2    o     E    5   4   3     1.206   119.761  -108.244  -0.61111
  11  H3    hn    E    8   5   4     1.006   118.708   169.131   0.30658
  12  C4    c     B    7   6   4     1.527   108.208   -33.617   0.71086
  13  C9A   ca    B    9   6   4     1.400   121.677   175.303   0.11121
  14  C1*   c3    3    9   6   4     1.488   120.112     0.762   0.19778
  15  N5    nh    B    7   6   4     1.481   114.953  -163.971  -0.70815
  16  H1*   h1    E   14   9   6     1.091   110.508   148.288   0.07396
  17  H1*   h1    E   14   9   6     1.094   109.531    34.132   0.07396
  18  H5    hn    E   15   7   6     1.013   116.643   172.266   0.35742
  19  C2*   c3    3   14   9   6     1.561   114.646   -86.525   0.07982
  20  C5A   ca    B   13   9   6     1.409   120.423    -8.092   0.09273
  21  C9    ca    B   13   9   6     1.406   122.728   171.775  -0.10129
  22  O4    o     E   12   7   6     1.219   123.320  -149.359  -0.58867
  23  H2*   h1    E   19  14   9     1.093   107.536    82.373   0.05788
  24  H9    ha    E   21  13   9     1.085   120.704    -6.259   0.15792
  25  C3*   c3    3   19  14   9     1.554   110.150  -159.554   0.21035
  26  C6    ca    B   20  13   9     1.390   121.067  -174.331  -0.16156
  27  C8    ca    B   21  13   9     1.398   121.928   175.156  -0.08564
  28  O2*   oh    S   19  14   9     1.451   113.188   -36.552  -0.67596
  29  H3*   h1    E   25  19  14     1.094   107.850  -175.639   0.04124
  30  H6    ha    E   26  20  13     1.081   119.439   178.084   0.15302
  31  HO2*  ho    E   28  19  14     1.001   112.324   176.349   0.43148
  32  C4*   c3    3   25  19  14     1.551   114.596   -53.688   0.02905
  33  C8M   c3    3   27  21  13     1.521   119.171  -179.325  -0.23499
  34  C7    ca    B   27  21  13     1.397   119.834     0.843  -0.07775
  35  O3*   oh    S   25  19  14     1.429   109.071    65.758  -0.66765
  36  H4*   h1    E   32  25  19     1.093   108.009    67.006   0.10010
  37  H8M   hc    E   33  27  21     1.092   111.003    -6.480   0.09610
  38  H8M   hc    E   33  27  21     1.091   110.679  -125.760   0.09610
  39  H8M   hc    E   33  27  21     1.090   110.882   113.348   0.09610
  40  HO3*  ho    E   35  25  19     1.006   104.478    80.406   0.44497
  41  C5*   c3    3   32  25  19     1.560   110.051  -176.957   0.32130
```

```
  42  C7M   c3    3   34  27  21     1.521   121.792  -179.041  -0.23423
  43  O4*   oh    S   32  25  19     1.445   111.849   -51.693  -0.71618
  44  H5*   h1    E   41  32  25     1.094   110.742   170.669   0.01987
  45  H5*   h1    E   41  32  25     1.093   108.127   -71.357   0.01987
  46  H7M   hc    E   42  34  27     1.090   111.521   172.730   0.09610
  47  H7M   hc    E   42  34  27     1.090   110.543    53.016   0.09610
  48  H7M   hc    E   42  34  27     1.091   110.254   -67.438   0.09610
  49  HO4*  ho    E   43  32  25     1.083   105.054   -93.601   0.51790
  50  O5*   os    S   41  32  25     1.450   112.449    46.991  -0.65813
  51  P     p5    3   50  41  32     1.559   117.661    83.091   1.56460
  52  O1P   o     E   51  50  41     1.460   110.288    86.188  -0.99869
  53  O2P   o     E   51  50  41     1.468   111.030  -152.815  -0.99869
  54  O3P   o     E   51  50  41     1.460   110.357   -33.185  -0.99869

LOOP CLOSING EXPLICIT
 N3   C4
 N5   C5A
 C6   C7

IMPROPER
 N1   C4A  C10  N10
 C2   C4   N3   H3
 N3   N1   C2   O2
 C4A  N3   C4   O4
 C5A  C4A  N5   H5
 C6   C9A  C5A  N5
 C7   C5A  C6   H6
 C7M  C6   C7   C8
 C8   C9A  C9   H9
 C8M  C7   C8   C9
 C9   C5A  C9A  N10
 C10  C1*  N10  C9A
```

2. Supplementary Figures

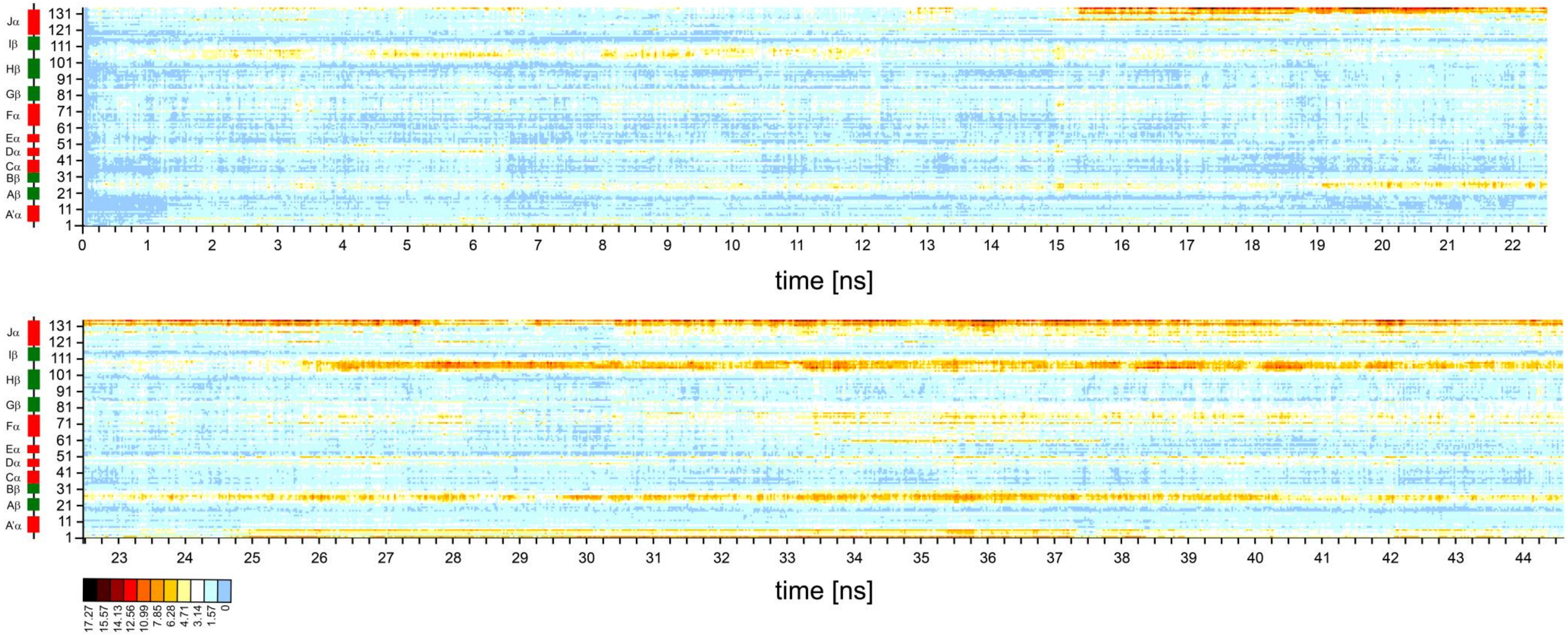

Supplementary Figure 1: Residue-resolved RMSD heatmap of the dark-state simulation. The trajectory was aligned over the backbone atoms of chain A and residue-wise RMSD values were calculated for chain B using the RMSF visualizer tool of VMD. The color scale corresponds depicts values between 0 (light-blue) and 17.27 Å (black). Secondary structure elements in topological order are depicted next to the residue numbers with α-helices in red and β-strands in green. The residue-resolved RMSD plot (Fig. 2. D, dark grey line) was derived from this data. by averaging over the RMSD values over the trajectory.

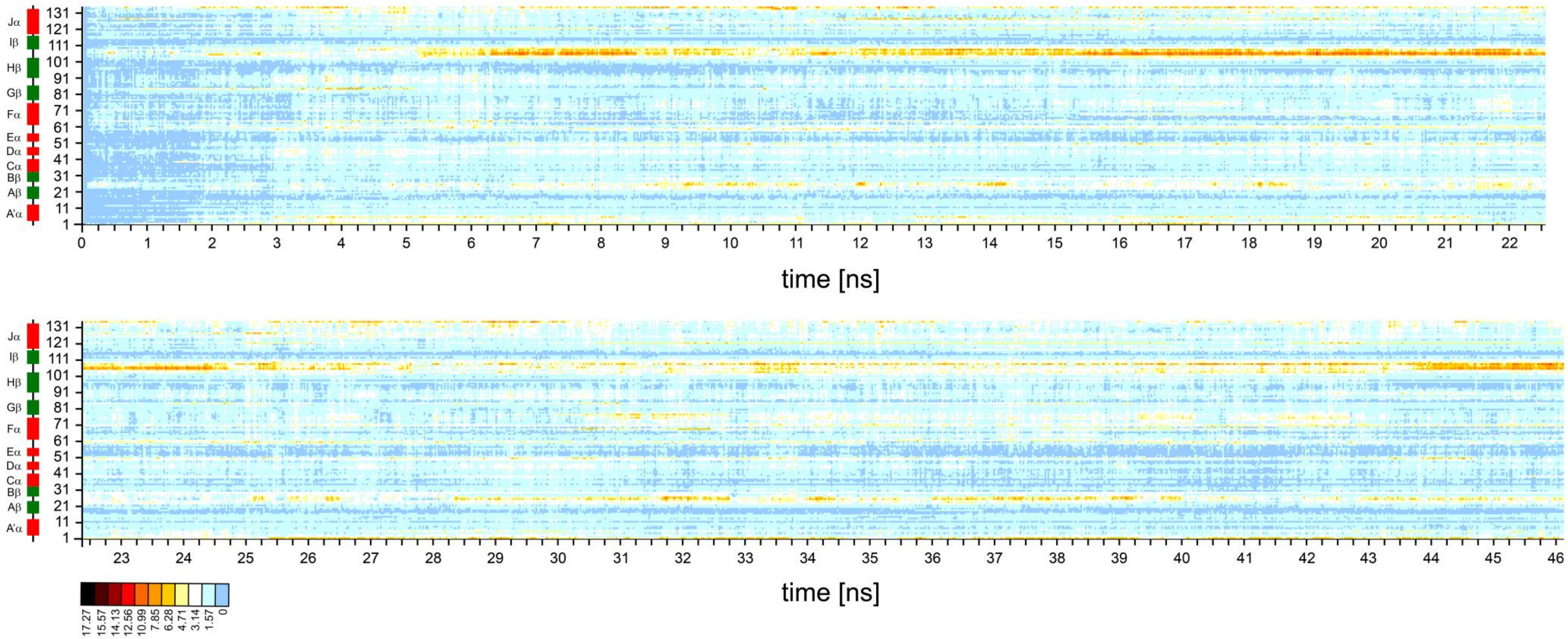

Supplementary Figure 2: Residue-resolved RMSD heatmap of the light-state simulation. The trajectory was aligned over the backbone atoms of chain A and residue-wise RMSD values were calculated for chain B using the RMSF visualizer tool of VMD. The color scale corresponds depicts values between 0 (light-blue) and 17.27 Å (black). Secondary structure elements in topological order are depicted next to the residue numbers with α-helices in red and β-strands in green. The residue-resolved RMSD plot (Fig. 2. D, cyan line) was derived from this data. by averaging over the RMSD values over the trajectory.

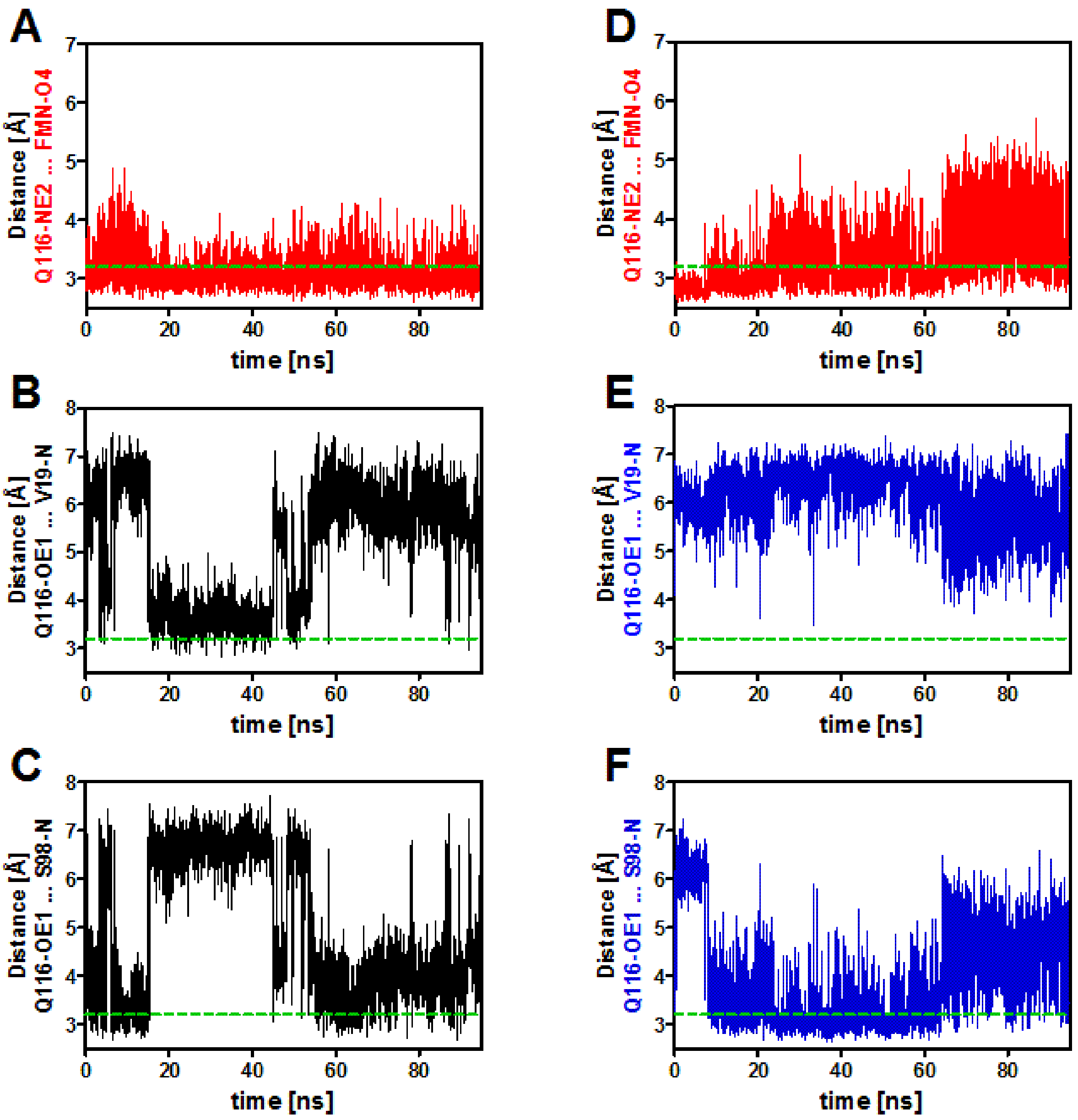

Supplementary Figure 3: Relevant distances between the side chain nitrogen and oxygen atoms of Q116 and the FMN chromophore (A, D) as well as to the backbone amide atoms of V19 and S98 (B, C,E,F) obtained from the 95 ns dark- and light-state trajectories (2D, 2L). On the left side (A, B, C) distances derived from the dark-state trajectory are shown, while on the right side (D, E, F) distances derived from the light-state trajectory are depicted. The dashed green line represents the 3.2 Å hydrogen bonding distance cut-off (Jeffrey, 1997, Steiner, 2002).

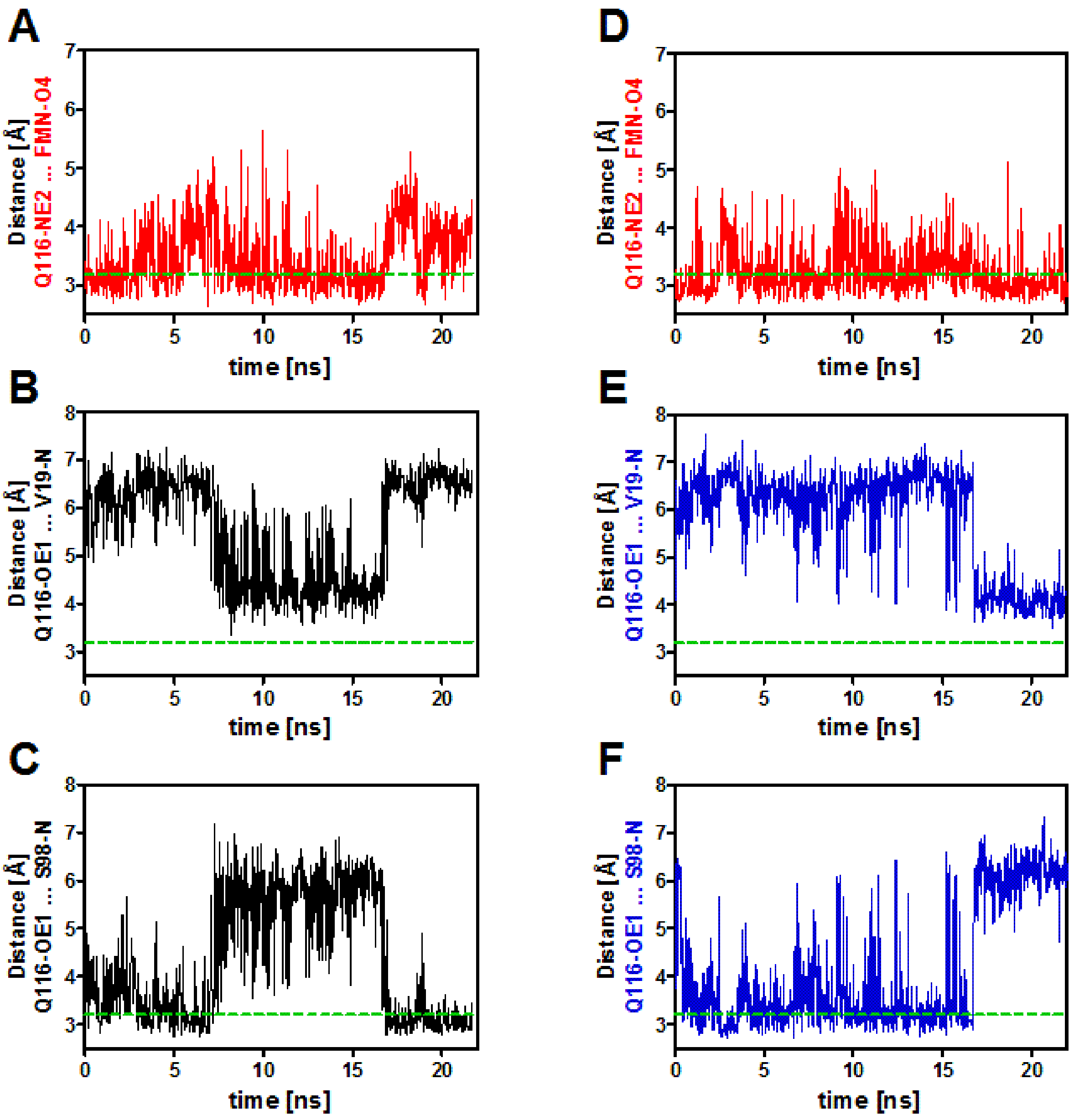

Supplementary Figure 4: Relevant distances between the side chain nitrogen and oxygen atoms of Q116 and the FMN chromophore (A, D) as well as to the backbone amide atoms of V19 and S98 (B, C, E, F) obtained from the 25 ns dark- and light-state trajectories (3D, 3L). On the left side (A, B, C) distances derived from the dark-state trajectory are shown, while on the right side (D, E, F) distances derived from the light-state trajectory are depicted. The dashed green line represents the 3.2 Å hydrogen bonding distance cut-off (Jeffrey, 1997, Steiner. 2002).

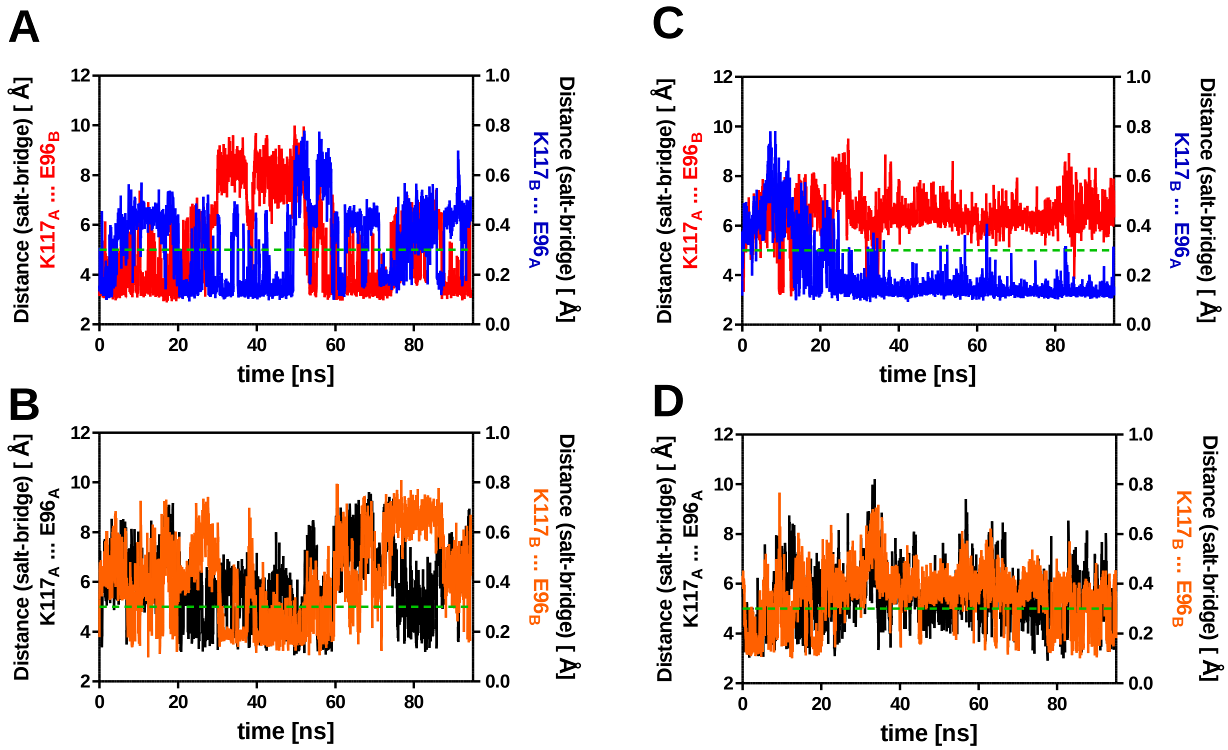

Supplementary Figure 5: Distance between residues constituting intra- and inter-subunit salt bridges derived from the 95 ns dark- and light-state trajectories (2D, 2L). (A) Distance between atoms that would constitute the inter-subunit K117-*E96'* salt-bridge in the dark state. (B) Distance between atoms constituting the intra-subunit K117-E96 salt-bridge in the dark state. (C) Distance between atoms that constitute the inter-subunit K117-*E96'* salt-bridge in the light state. (D) Distance between atoms that would constitute the intra-subunit K117-E96 salt-bridge in the light state. The dashed green line depicts the distance cut-off used for salt-bridge identification (see Materials and Methods for details)

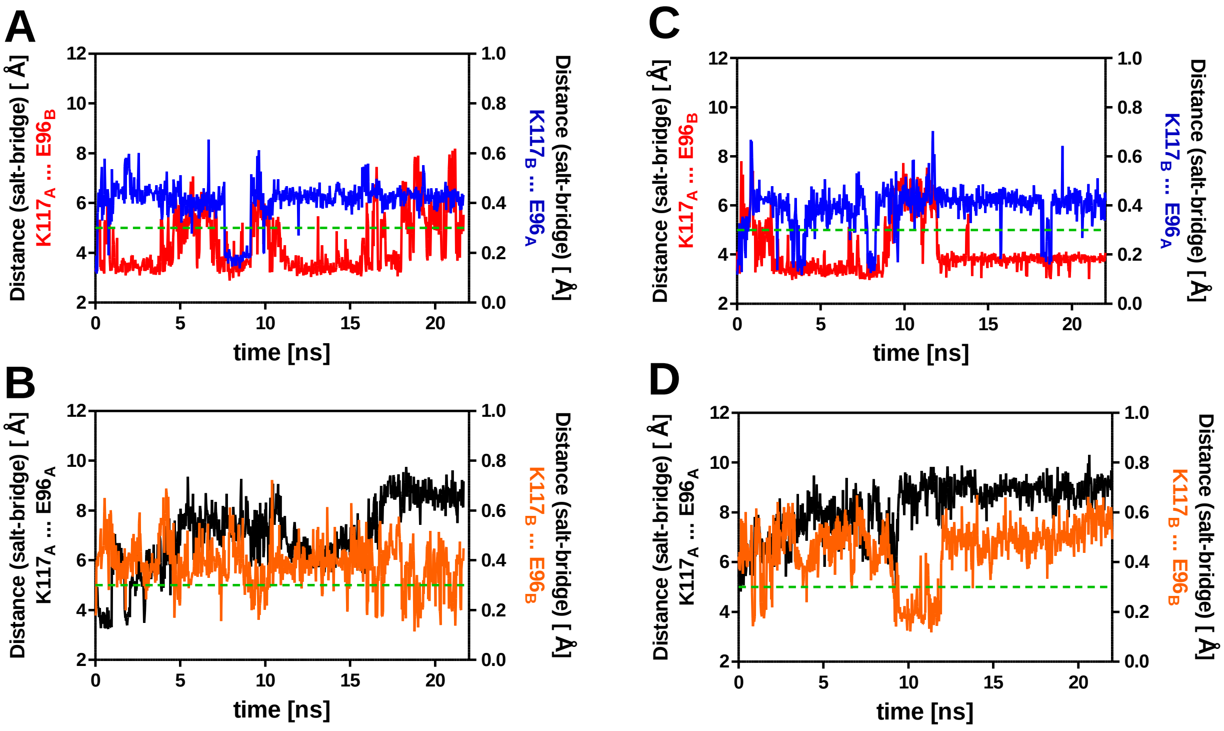

Supplementary Figure 6: Distance between residues constituting intra- and inter-subunit salt bridges derived from the 25 ns dark- and light-state trajectories (3D, 3L). (A) Distance between atoms that would constitute the inter-subunit K117-*E96'* salt-bridge in the dark state. (B) Distance between atoms constituting the intra-subunit K117-E96 salt-bridge in the dark state. (C) Distance between atoms that constitute the inter-subunit K117-*E96'* salt-bridge in the light state. (D) Distance between atoms that would constitute the intra-subunit K117-E96 salt-bridge in the light state. The dashed green line depicts the distance cut-off used for salt-bridge identification (see Materials and Methods for details)

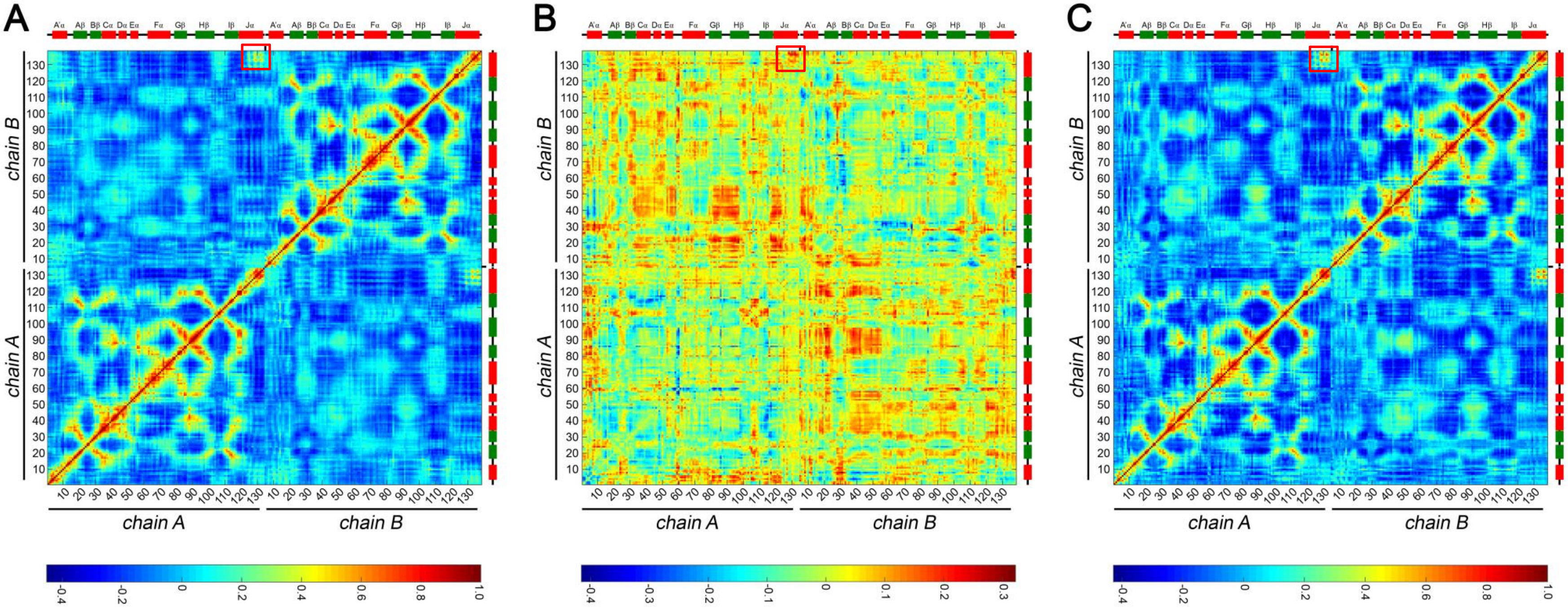

Supplementary Figure 7: Dynamic cross-correlation analysis matrices for all dark- (A) and light-state MD trajectories (C). For each plot, the three dark- and light-state cross-correlation matrices were combined. Positive values and negative values are indicative for correlated- and anti-correlated motions. respectively. The middle panel (B) shows the corresponding light-dark cross-correlation data obtained my subtraction of the respective average light- and dark-state dynamic cross-correlation matrices. Please note that, positive values in the light-dark plot do not necessarily indicate increased correlated motions in the light-state as observed for the cross-correlation between the Jα-helices of the two subunits (red box) as they could also result from e.g. increased anti-correlated motions in the dark state. The light-dark plot thus merely highlights structural regions showing differences in correlated or anti-correlated motions between the simulations.

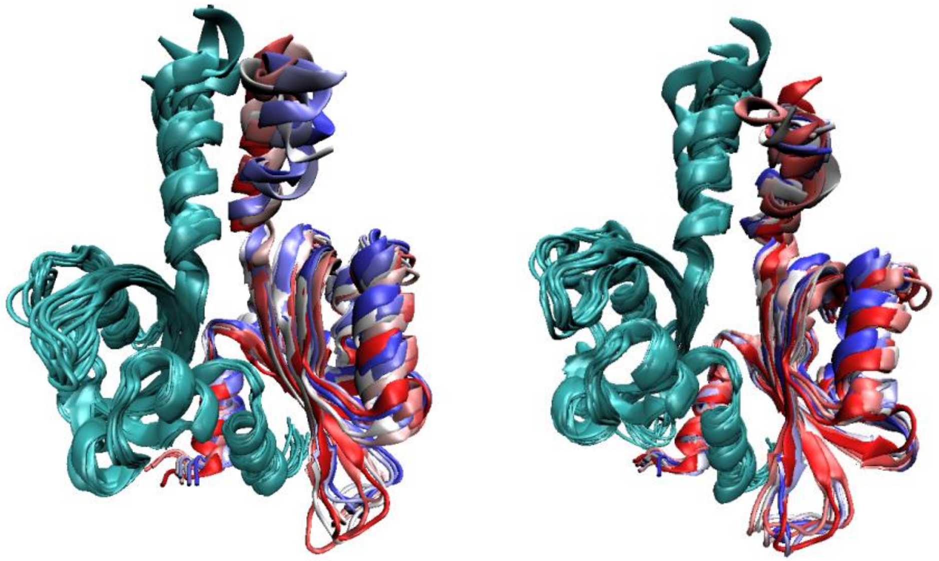

Supplementary Figure 8: Superimposition of two independent 150 ns Amber99 trajectories for the PpSB1-LOV light-state, illustrating the known destabilization bias of this class of force fields. In the corresponding dark-state simulations similar behaviour was observed. In both panels chain B is color coded by simulation time (t=0: dark red; t=150 ns: dark blue). The Jα helix depicted on top of the figure is destabilized within the first 15 ns (light red) and stayed broken over the whole simulation time.

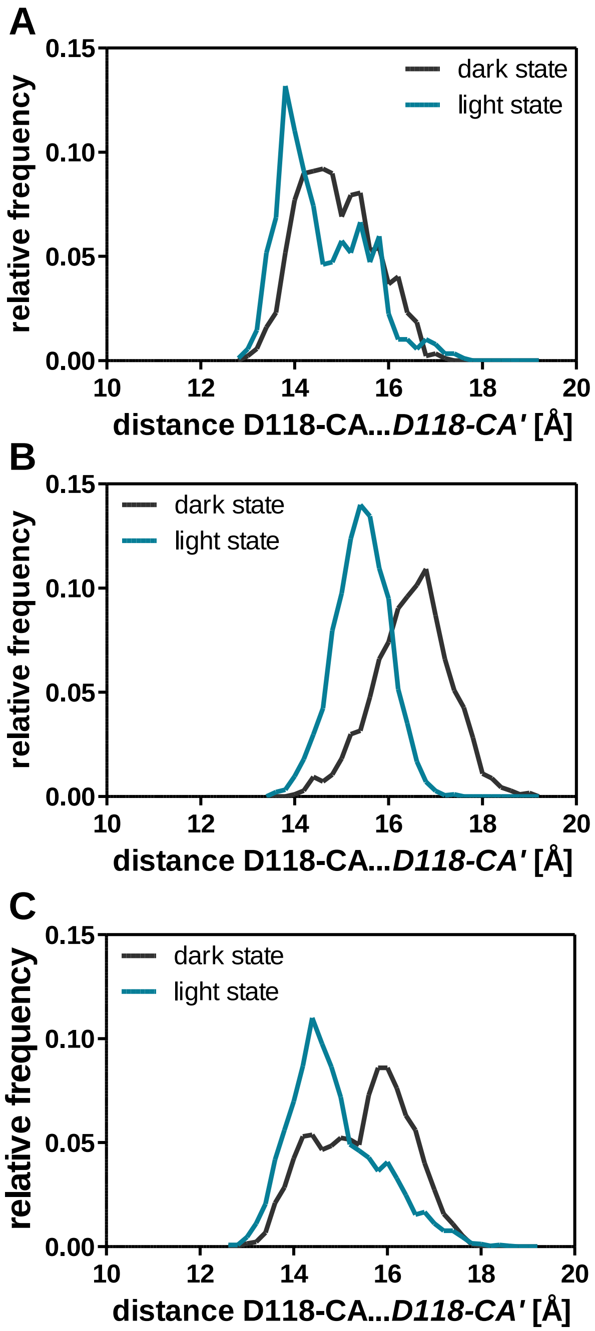

Supplemetary Figure 9: Illustration of Iβ-tilting in all dark- and light-state simulations. To illustrate tilting of the C-terminal end of the Iβ strand in the light-state relative to the dark-state simulations, the inter-subunit distance between the CA atoms of D118, a proximal residue at the C-terminal end of the Iβ strand, was measured. The corresponding distances derived from all dark-state (dark grey line) and light-state (cyan line) simulations are plotted as frequency distribution for the two short trajectories (A: 1D, 1L; B: 2D, 2L) and the longer trajectories (C: 3D, 3L). A similar trend is observed in all simulations. The mean distance between the two subunits is lower in the light-state simulations compared to the corresponding dark-state simulations, while the distance between the CA atoms of G114 (centrally located on Iβ) between the two subunits, stays constant over the trajectories (data not shown). This indicates that Iβ (i.e. the C-terminal end where K117 is located) is tilting away from the core domain towards the dimer interface in the light-state compared to the dark state. This displacement can account for stabilization of the E96-K117 salt-bridge in the light-state.
